# Proper regulation of inositolphosphorylceramide levels is required for acquirement of low pH resistance in budding yeast

**DOI:** 10.1038/s41598-020-67734-8

**Published:** 2020-07-01

**Authors:** Mikiko Otsu, Moeko Toume, Yutaro Yamaguchi, Motohiro Tani

**Affiliations:** Department of Chemistry, Faculty of Sciences, Kyushu University, 744, Motooka, Nishi-ku, Fukuoka, 819-0395 Japan

**Keywords:** Biochemistry, Genetics, Microbiology

## Abstract

All organisms have stress response systems to protect themselves from various environmental stresses, and regulation of membrane lipids is thought to play an important role in acquirement of stress tolerance. Complex sphingolipids in the yeast *Saccharomyces cerevisiae* are classified into three types based on differences in the structure of the polar head group, and the compositions and quantities of complex sphingolipids in biomembranes are tightly regulated. In this study, we found that the accumulation of inositol phosphorylceramides (IPCs) due to a defect of mannosylinositol phosphorylceramide biosynthesis (*sur1∆ csh1∆*), i.e., disruption of the balance of the composition of complex sphingolipids, causes hypersensitivity to low pH conditions (pH 4.0–2.5). Furthermore, screening of suppressor mutations that confer low pH resistance to *sur1∆ csh1∆* cells revealed that a change in ergosterol homeostasis at plasma membranes can rescue the hypersensitivity, suggesting the functional relationship between complex sphingolipids and ergosterol under low pH conditions. Under low pH conditions, wild-type yeast cells exhibited decreases in IPC levels, and forced enhancement of the biosynthesis of IPCs causes low pH hypersensitivity. Thus, it was suggested that the accumulation of IPCs is detrimental to yeast under low pH conditions, and downregulation of IPC levels is one of the adaptation mechanisms for low pH conditions.

## Introduction

All organisms are exposed to various stresses caused by changing environmental factors, such as temperature, osmotic pressure, pH, nutritional status and chemicals, and thus have various stress response systems to protect themselves^1^. Sphingolipids, one of the major components of biomembranes of eukaryotic cells, are thought to play important roles in acquirement of resistance against some environmental stresses. For example, in the budding yeast *Saccharomyces cerevisiae*, the biosynthesis of sphingolipids is upregulated during heat stress, which is essential for the acquirement of thermoresistance^2^. Complex sphingolipids, which each comprise a polar head group and a ceramide (Cer) composed of a fatty acid and a LCB, form lipid microdomains together with sterols in eukaryotic biomembranes^3,4^. Several proteins involved in acquirement of stress resistance are associated with lipid microdomains in yeast, and thus disruption of the structure of lipid microdomains, which is induced by inhibition of the biosynthesis of complex sphingolipids and sterols, causes mislocalization and dysregulation of these proteins, and can impair the resistance to several stresses^5–7^. In addition, it is suggested that complex sphingolipids also contribute to maintenance of the physical properties of plasma membranes including membrane fluidity and thickness^8,9^, which may also influence stress resistance.

**Figure 1:**
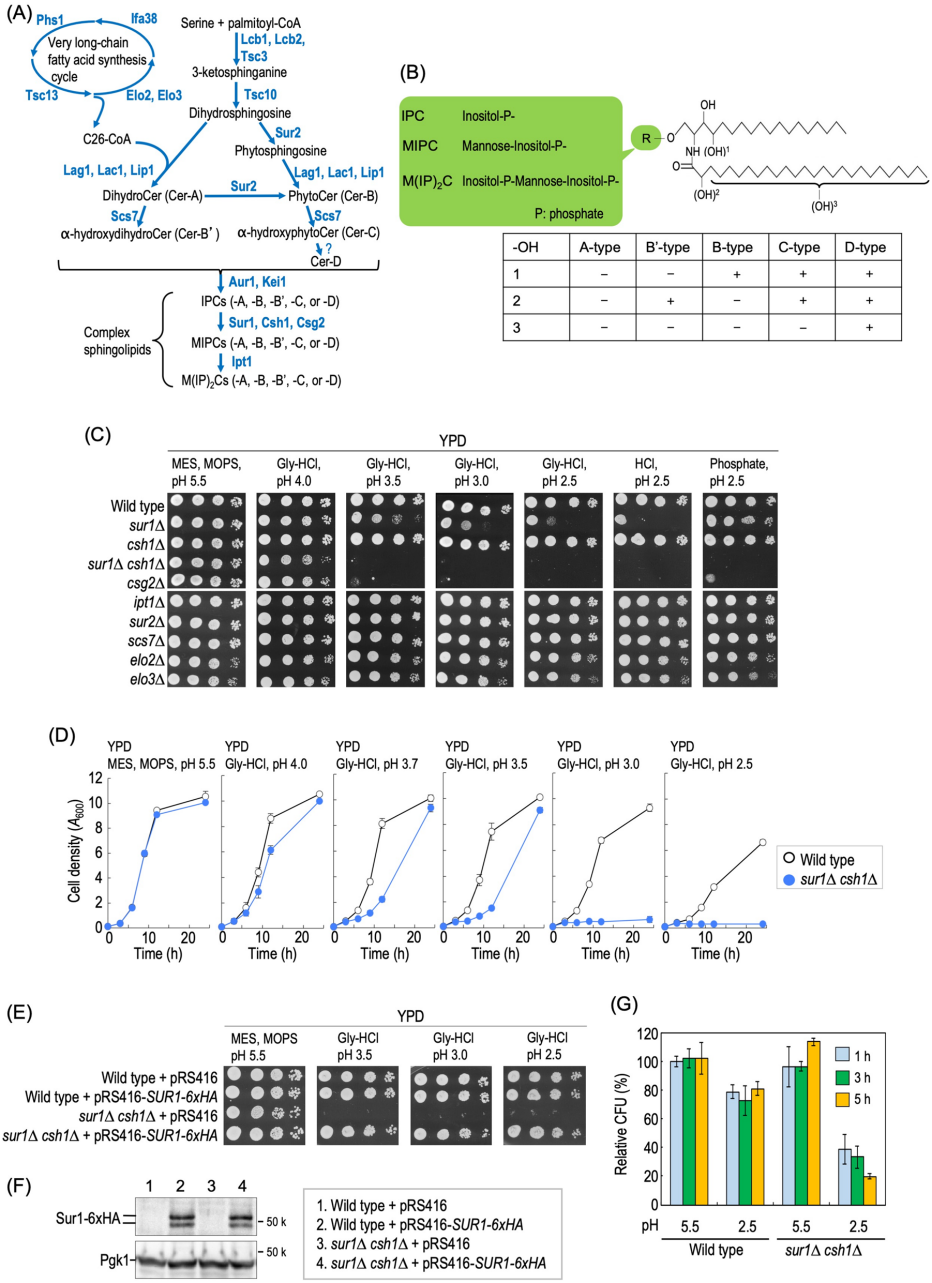
MIPC biosynthesis-deficient cells exhibit hypersensitivity to extracellular low pH. (**A**) Complex sphingolipid biosynthesis pathway of yeast *Saccharomyces cerevisiae*. The pathway and proteins responsible for the synthesis of complex sphingolipids in *S. cerevisiae* are shown. (**B**) Structures of *S. cerevisiae* complex sphingolipids. *S. cerevisiae* complex sphingolipids have three types of polar head group, and can be divided into IPC, MIPC, and M(IP)_2_C. The sites labelled 1, 2 and 3 in the Cer moiety are hydroxylated by Sur2, Scs7, and an unidentified hydroxylase(s), respectively. Sites 1 and 2 are at the C-4 position of the LCBs and the C-2 position of the very long-chain fatty acids, respectively. Site 3 is also on the very long-chain fatty acids, but the position has not been determined. (**C**) Cells cultured in YPD medium were spotted onto agar plates containing YPD medium buffered to the indicated pH, and then incubated at 30 °C for 2 days. The details are given under METHODS. (**D**) Time course of cell growth. Cells were cultured overnight in YPD medium at 30 °C and then diluted (0.1 *A*_600_ units/ml) in fresh YPD medium buffered to the indicated pH, and then aliquots of cell suspensions were subjected to cell density measurements (*A*_600_) at the indicated times. Data represent means ± SD for one experiment (triplicate) representative of three independent experiments. (**E**) Cells harboring pRS416 (empty vector) or pRS416-*SUR1-6xHA* were cultured overnight in SC-Ura (pH 6.0), spotted onto agar plates containing YPD medium buffered to the indicated pH, and then incubated at 30 °C for 2 days. (**F**) Cells harboring pRS416 or pRS416-*SUR1-6xHA* were cultured overnight in SC-Ura (pH 6.0), and the cell extracts were immunoblotted using anti-HA or anti-Pgk1 antibodies. Sur1-6xHA gave two bands, the upper band being the *N*-glycosylated form^78^. Full Western blots are shown in Supplementary Fig. S10, *panel a*. (**G**) Cell viability under low pH conditions. Relative colony forming units (CFU) of wild-type and *sur1∆ csh1∆* cells exposed to YPD medium buffered to pH 5.5 or 2.5 for the indicate times were calculated as described under METHODS. Data represent means ± SD for one experiment (triplicate) representative of three independent experiments.

In *S. cerevisiae,* according to differences in the polar head group structure, complex sphingolipids are classified into three types, inositol phosphorylceramide (IPC), mannosylinositol phosphorylceramide (MIPC), and mannosyldiinositol phosphorylceramide (M(IP)_2_C)^3^ (Fig. 1A,B). In addition, the Cer moiety in yeast complex sphingolipids can be divided in five types (A, B, B′, C and D) according to the hydroxylation state^3,10^ (Fig. 1A,B). Deletion of the genes of MIPC synthase (*SUR1* and *CSH1*) or their regulatory subunit (*CSG2*) results in complete loss or drastic reduction of MIPCs and M(IP)_2_Cs, and accumulation of IPCs^11^. A defect of MIPC biosynthesis has deleterious effects under some stressful conditions. For instance, MIPC biosynthesis-deficient mutants exhibit hypersensitivity to exogenous Ca^2+^ and reduction of the rate of cell survival under nitrogen starvation^12–14^. The growth defects caused by these stressful conditions are suppressed by inhibition of biosynthesis of hydroxylated IPCs, suggesting that these phenotypes are not due to loss of MIPCs themselves but to the accumulation of hydroxylated IPCs^13,15^. *sur1∆ csh1∆* cells also exhibit impairment of cell wall integrity; however, the cell wall-defective phenotype is caused by loss of MIPCs themselves but not by accumulation of IPCs^16^. The biosynthesis of MIPCs is also related with the function and metabolism of glycerophospholipids; that is, the double defect of biosynthesis of phosphatidylserine and MIPC causes a strong growth defect and impairment of a specific vesicular trafficking pathway^17,18^. In addition, MIPCs are involved in maintenance of the asymmetry of glycerophospholipids at plasma membranes through regulation of glycerophospholipid flippases-regulating kinase, Fpk1^19^.

In *S. cerevisiae,* C-type complex sphingolipids are the most predominant species, and the levels of IPCs and M(IP)_2_Cs are much higher than that of MIPCs^10^. However, several lines of evidence have indicated that the compositions and quantities of complex sphingolipids in yeast biomembranes change under certain stressful conditions. *S. cerevisiae* and *Zygosaccharomyces bailii* exhibit increases in the IPC, MIPC and M(IP)_2_C levels in response to acetic acid stress^20^. Furthermore, the Target of Rapamycin (TOR) Complex 2 (TORC2)- and Ypk1-mediated signaling pathway, which upregulates sphingolipid biosynthesis, plays an important role in acquisition of resistance to acetic acid stress in *S. cerevisiae*^21^. Deletion of vacuolar H^+^-ATPase (V-ATPase), which causes a defect of vacuolar proton homeostasis, causes dramatic alteration of the complex sphingolipid composition including increases in MIPC levels, and it is suggested that the alteration is an adaptation mechanism against a defect of V-ATPase^22^. These results suggest that regulation of the compositions and quantities of complex sphingolipids in biomembranes is important for the adaptation of cells to certain stressful conditions.

In this study, we found that accumulation of IPCs due to a defect of MIPC biosynthesis causes a strong growth defect when the pH of the extracellular milieu become acidic. Furthermore, cellular IPC levels rapidly decreased under low pH conditions, and enhancement of the biosynthesis of IPCs caused low pH hypersensitivity, suggesting that decreases in IPC levels are one of the adaptation mechanisms for acquisition of low pH resistance. These findings provide new information as to the importance of regulation of complex sphingolipid levels under stressful conditions.

## Results

### Loss of MIPC biosynthesis causes hypersensitivity to extracellular low pH

To investigate whether or not an aberrant complex sphingolipid composition affects cell growth under low pH conditions, yeast cells lacking non-essential sphingolipid-metabolizing enzyme genes were used. The structural diversity of complex sphingolipids in the budding yeast is created by sphingolipid-metabolizing enzymes, *SUR2*, *SCS7*, *IPT1*, *SUR1*, and *CSH1* (Fig. 1A,B)^10^. In addition, cells lacking *ELO2* or *ELO3*, which are involved in biosynthesis of very-long chain fatty acids incorporated into yeast sphingolipids (Fig. 1A)^23^, were also used in the experiments. Although a delay of growth of wild-type cells was observed after 1-day culture on YPD plates buffered to pH 2.5 (Supplementary Fig. S1), the growth patterns on YPD plates buffered to pH 5.5 and pH 2.5 were nearly indistinguishable after 2 days culture (Fig. 1C). *sur1∆*, *csg2∆*, and *sur1∆ csh1∆* cells exhibited a strong growth defect on YPD plates buffered to pH 3.5, 3.0, and 2.5 with glycine–HCl, the most robust growth defect being observed in *sur1∆ csh1∆* cells (Fig. 1C). The low pH hypersensitivity of *sur1∆* and *csg2∆* cells coincided with the results of high-throughput screening^24,25^. In contrast, deletion of *IPT1* encoding M(IP)_2_C synthase did not confer the hypersensitivity (Fig. 1C). The low pH hypersensitivity of MIPC biosynthesis-deficient cells was also confirmed when pH 2.5 YPD plates were prepared by the addition of phospholic acid-sodium dihydrogen phosphate or HCl, indicating that the hypersensitivity does not depend on the means of adjusting the pH of the medium (Fig. 1C). The hypersensitivity to low pH conditions of *sur1∆ csh1∆* cells was also observed in liquid culture (Fig. 1D). The low pH hypersensitivity was restored by the expression of Sur1-6xHA^22^ in *sur1∆ csh1∆* cells (revertant) (Fig. 1E,F). When cells were incubated at pH 2.5, the reduction in cell viability of *sur1∆ csh1∆* cells was much more severe than that of wild-type cells, indicating that loss of MIPC biosynthesis causes cell death under low pH conditions (Fig. 1G).

### Low pH hypersensitivity of *sur1∆ csh1∆* cells is not due to intracellular acidification

**Figure 2:**
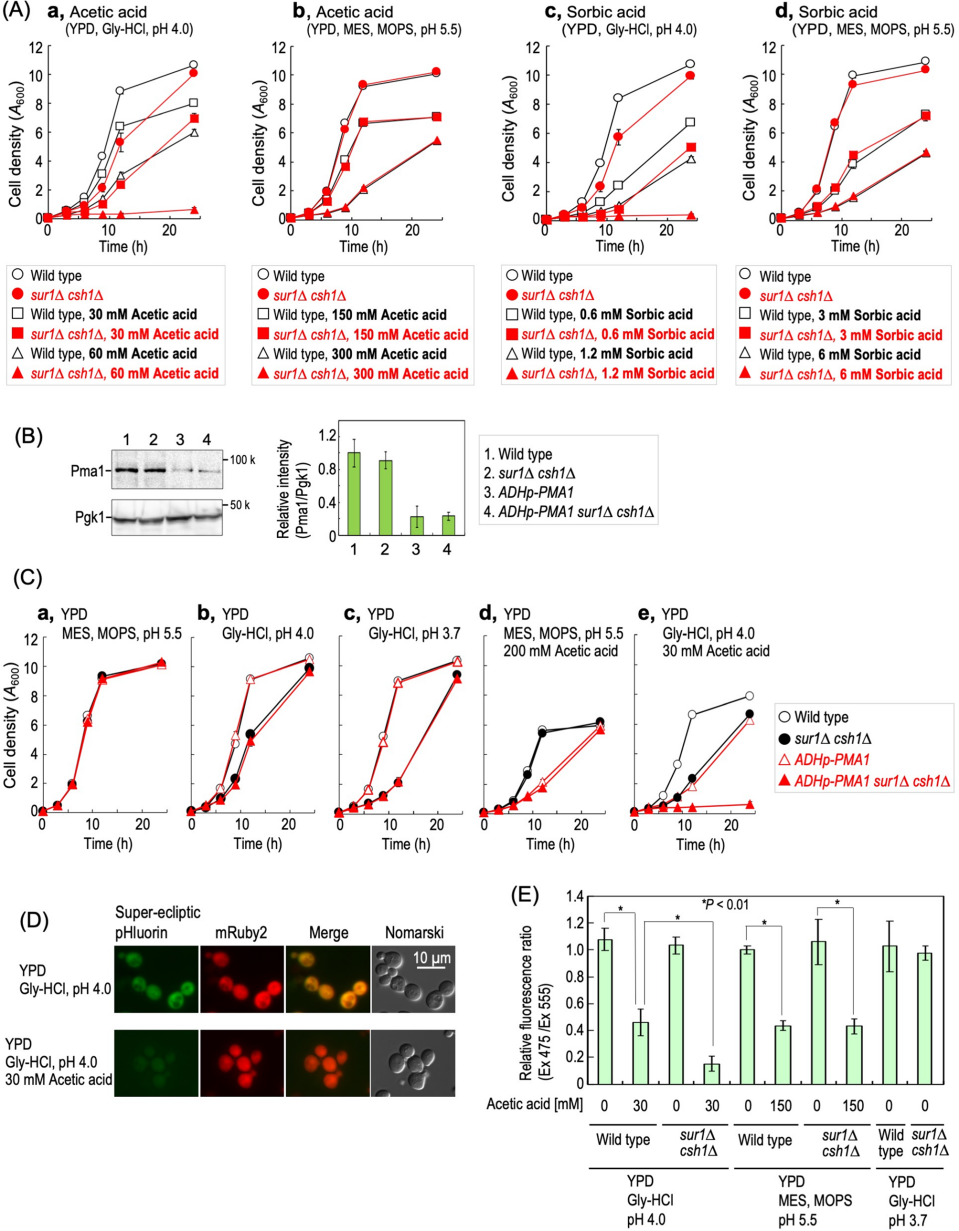
Low pH hypersensitivity of *sur1∆ csh1∆* cells is not caused by intracellular acidification. (**A**) Sensitivity to organic acids. Cells were cultured overnight in YPD medium at 30 °C and then diluted (0.1 *A*_600_ units/ml) in fresh YPD medium with or without the indicated concentrations of acetic acid or sorbic acid, which was buffered to pH 4.0 or 5.5, and then cultured for the indicated times. Aliquots of the cell suspensions were subjected to cell density measurements (*A*_600_) at the indicated times. (**B**) Cells expressing Pma1 with the native promoter or *ADH* promoter were grown to the exponential phase, and the cell extracts were immunoblotted using anti-Pma1 or anti-Pgk1. Full Western blots are shown in Supplementary Fig. S10, *panel b*. (**C**) Effects of repression of Pma1 expression on sensitivity to acetic acid and low pH conditions. Cells were cultured overnight in YPD medium at 30 °C and then diluted (0.1 *A*_600_ units/ml) in fresh YPD medium with or without the indicated concentration of acetic acid, which was buffered to the indicated pH, and then cultured for the indicated times. Data represent means ± SD for one experiment (triplicate) representative of three independent experiments. (**D**) Wild-type cells harboring pKL06 were exposed for 1 h to YPD medium with or without 30 mM acetic acid, which was buffered to pH 4.0, as described under METHODS. The cells were viewed under a fluorescence microscope. (**E**) Wild-type and *sur1∆ csh1∆* cells harboring pKL06 were exposed for 1 h to the indicated media and viewed under a fluorescence microscope. The graph indicates the ratio of fluorescence intensity of super*-*ecliptic pHluorin to that of mRuby2 in individual cells. The value of wild-type cells incubated at pH 5.5 was taken as 1. Data represent means ± SEM (100 cells for each experimental condition) in one experiment representative of three independent experiments. The details are given under METHODS.

The intracellular pH is tightly maintained around neutral, but is slightly affected by the extracellular pH^26^. Therefore, it is important to investigate whether or not the low pH hypersensitivity of *sur1∆ csh1∆* cells is caused by intracellular acidification. When yeast cells are exposed to undissociated organic acids, the organic acids permeabilize into plasma membranes, and subsequently acidify the cytosolic region after dissociation into protons and anions^27^. Thus, we examined the sensitivities to acetic acid and sorbic acid, typical inducers of organic acid stress. When the pH value of the culture medium is lower than the pKa value of acetic acid and sorbic acid (approx. 4.76), they can effectively be incorporated into cells^27^. Thus, we firstly examined the effects of the organic acids at pH 4.0 (Fig. 2A). At pH 4.0, the delay of growth of *sur1∆ csh1∆* cells due to addition of acetic acid or sorbic acid was more severe than that of wild-type cells (Fig. 2A, *panels a* and *c*). Since *sur1∆ csh1∆* cells exhibited delay of cell growth at pH 4.0 even in the absence of organic acids, it is possible that low pH conditions and the presence of organic acids had a synthetic effect on the growth of *sur1∆ csh1∆* cells, so we next examined the effect of the organic acids at pH 5.5. It was reported that intracellular acidification occurs on the addition of acetic acid at pH 5.5; however, the effect of acetic acid at pH 5.5 is weaker than that at the lower pH^28^. Although at pH 5.5 a five-fold concentration of acetic acid or sorbic acid was required for induction of the growth inhibition observed at pH 4.0, no difference in the growth inhibition pattern was observed between wild-type and *sur1∆ csh1∆* cells (Fig. 2A, *panels b* and *d*).

The intracellularly accumulated protons due to organic acid stress can be excluded from the cytosol by the plasma membrane H^+^-ATPase (Pma1)^29^. Thus, we next investigated the effect of repression of Pma1 expression. Replacement of the promoter region of chromosomal *PMA1* with a ADH promoter (*ADHp-PMA1*) resulted in a decrease in the expression level of Pma1 (Fig. 2B). The repression of Pma1 expression did not affect cell growth at pH 5.5 (Fig. 2C, *panel a*). As expected, in both *sur1∆ csh1∆* and *SUR1 CSH1* cells, the repression of Pma1 expression enhanced the growth inhibition caused by the addition of 200 or 30 mM acetic acid at pH 5.5 or 4.0 (Fig. 2C, *panels d* and *e*), probably due to a delay of exclusion of the intracellularly accumulated protons. In contrast, the repression of Pma1 expression did not enhance the slow growth of *SUR1*- and *CSH1*-deleted cells at pH 4.0 and 3.7 (Fig. 2C, *panels b* and *c*). Thus, it was suggested that the low pH hypersensitivity of *sur1∆ csh1∆* cells is caused by the change of pH in the extracellular milieu but not by intracellular acidification. To monitor intracellular acidification, a fusion protein (pHluorin-mRuby2) that contains super*-*ecliptic pHluorin (a pH-sensitive GFP variation) and mRuby2 (a pH-stable RFP) was used^30,31^. As shown in Fig. 2D, when wild-type cells expressing pHluorin-mRuby2 were exposed to 30 mM acetic acid at pH 4.0, a decrease in super*-*ecliptic pHluorin fluorescence, but not in mRuby2 fluorescence, was observed, implying intracellular acidification due to the treatment with acetic acid. Figure 2E shows results as to the ratio of super*-*ecliptic pHluorin to mRuby2 fluorescence in wild-type and *sur1∆ csh1∆* cells under various conditions. At pH 4.0, the effect of 30 mM acetic acid on intracellular acidification of *sur1∆ csh1∆* cells was more severe than that of wild-type cells (Fig. 2E). In contrast, at pH 5.5, the effect of 150 mM acetic acid did not differ between wild-type and *sur1∆ csh1∆* cells (Fig. 2E). These results coincided the results of acetic acid sensitivity of wild-type and *sur1∆ csh1∆* cells at pH 4.0 and pH 5.5 (Fig. 2A, *panels a* and *b*). On the other hand, incubation of wild-type and *sur1∆ csh1∆* cells at pH 3.7 did not have a notable effect on the intracellular acidification (Fig. 2E), suggesting that pH 3.7, a culture condition causing a dramatic delay of growth of *sur1∆ csh1∆* cells (Figs. 1D, 2C), does not cause intracellular acidification under our experimental conditions. This supports the notion that the low pH hypersensitivity of *sur1∆ csh1∆* cells does not related to intracellular acidification.

### Permeability of plasma membranes is increased in *sur1∆ csh1∆* cells under low pH conditions

**Figure 3:**
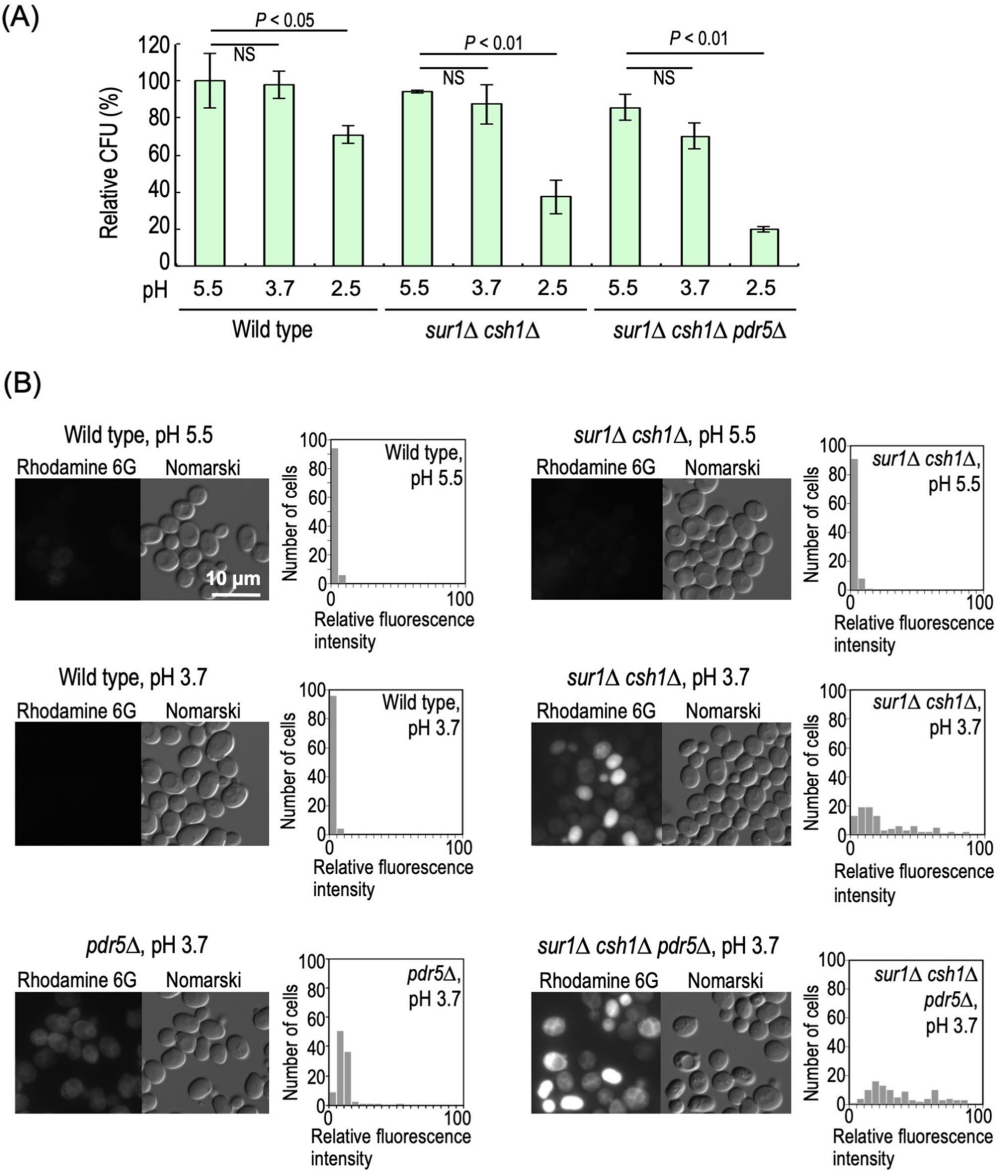
Plasma membrane permeability of *sur1∆ csh1∆* cells under low pH conditions. (**A**) Cell viability of wild-type, *sur1∆ csh1∆*, and *sur1∆ csh1∆ pdr5∆* cells under low pH conditions. Relative colony forming units (CFU) of cells exposed to YPD medium buffered to pH 5.5, 3.7, or 2.5 for 2 h were calculated as described under METHODS. Data represent means ± SD for one experiment (triplicate) representative of three independent experiments. NS, no significant difference. (**B**) Efficiency of incorporation of rhodamine 6G into cells incubated at pH 5.5 or 3.7. The graphs indicate the frequency distributions of rhodamine 6G fluorescence intensity in individual cells. Data represent the values for 100 cells for individual strains. The details are given under METHODS.

The hypersensitivity of *sur1∆ csh1∆* cells to extracellular acidification prompted us to investigate plasma membrane integrity. The permeability of plasma membranes was evaluated as to the efficiency of incorporation of a lipofilic fluorescent dye, rhodamine 6G^32,33^ (Fig. 3B). Since an increase in plasma membrane permeability is observed in dead cells due to non-specific effects, we firstly determined experimental conditions under which the viability of *sur1∆ csh1∆* cells is not drastically reduced even under low pH conditions. The viability of *sur1∆ csh1∆* cells did not significantly decrease on 2-h incubation at pH 3.7 (but not at pH 2.5) (Fig. 3A), culture conditions causing a delay of cell growth (Fig. 1D). Thus, we decided to evaluate the plasma-membrane permeability at pH 3.7. In both wild-type and *sur1∆ csh1∆* cells, intracellular accumulation of rhodamine 6G was hardly observed at pH 5.5; however, an increase in the accumulation was observed when *sur1∆ csh1∆* cells, but not wild-type cells, were incubated at pH 3.7 for 2 h (Fig. 3B). The accumulation of rhodamine 6G was also observed when *sur1∆ csh1∆* cells were incubated at pH 4.0 (data not shown), a culture condition used for organic acid sensitivity (Fig. 2A). Intracellularly accumulated rhodamine 6G is extruded by a multidrug efflux transporter, Pdr5^32,33^, and thus there is a possibility that accumulation of this dye in *sur1∆ csh1∆* cells cultured under low pH conditions is caused by a functional defect of Pdr5. The accumulation of rhodamine 6G in *pdr5∆* cells was much higher than that in wild-type cells, as reported previously^32,33^, and enhancement of the accumulation caused by deletion of *SUR1* and *CSH1* was also observed in *PDR5*-deleted cells (*pdr5∆* versus *sur1∆ csh1∆ pdr5∆* cells) (Fig. 3B), indicating that plasma membrane permeability is increased in *sur1∆ csh1∆* cells cultured under low pH conditions.

### Screening of suppressor mutations that rescue the low pH hypersensitivity of *sur1∆ csh1∆* cells

**Figure 4:**
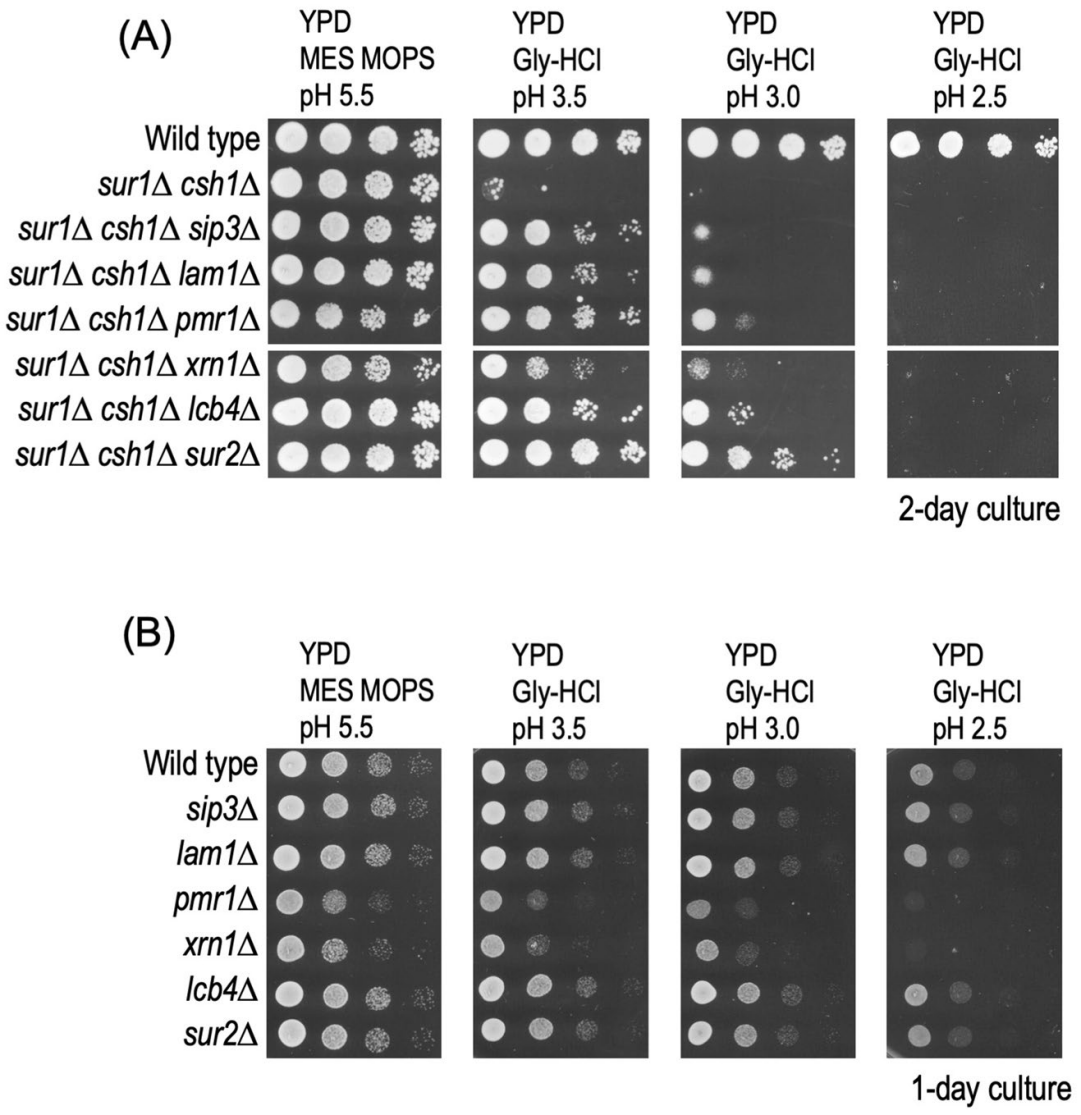
Suppressor mutations that confer resistance to low pH conditions in *sur1∆ csh1∆* cells. Effects of deletion of genes identified on transposon mutagenesis screening on the low pH hypersensitivity of *sur1∆ csh1∆* cells. Deletion of each gene in *sur1∆ csh1∆* (**A**) or wild-type (**B**) cells was performed by replacing the entire open reading frame with the antibiotic-resistance cassette. Cells cultured in YPD medium were spotted onto agar plates containing YPD medium buffered to the indicated pH, and then incubated at 30 °C for 2 days (**A**) or 1 day (**B**). The details are given under METHODS.

To gain more mechanistic insight into how a defect of MIPC biosynthesis induces low pH hypersensitivity, we screened for suppressor mutations that rescue the hypersensitivity. The chromosomes of *sur1∆ csh1∆* cells were randomly mutated by insertion of the *mTn-lacZ:LEU2* transposon^34^, and then mutant cells that can grow well on SC-Leu plates buffered to pH 3.7 were isolated. From ∼50,000 transformants, finally, we identified six mutations (*sip3*, *lam1*, *pmr1*, *xrn1*, *lcb4*, and *sur2*) that rescue the low pH hypersensitivity of *sur1∆ csh1∆* cells (Supplementary Table S1). Figure 4A shows suppression of the low pH hypersensitivity of *sur1∆ csh1∆* cells on deletion of the entire open reading frames of these genes. In contrast, the deletion of these genes did not have a suppressive effect on wild-type cells (Fig. 4B), indicating that these suppressor mutations are only effective in *sur1∆ csh1∆* cells.

### Accumulation of IPCs is causative of the low pH hypersensitivity of *sur1∆ csh1∆* cells

**Figure 5:**
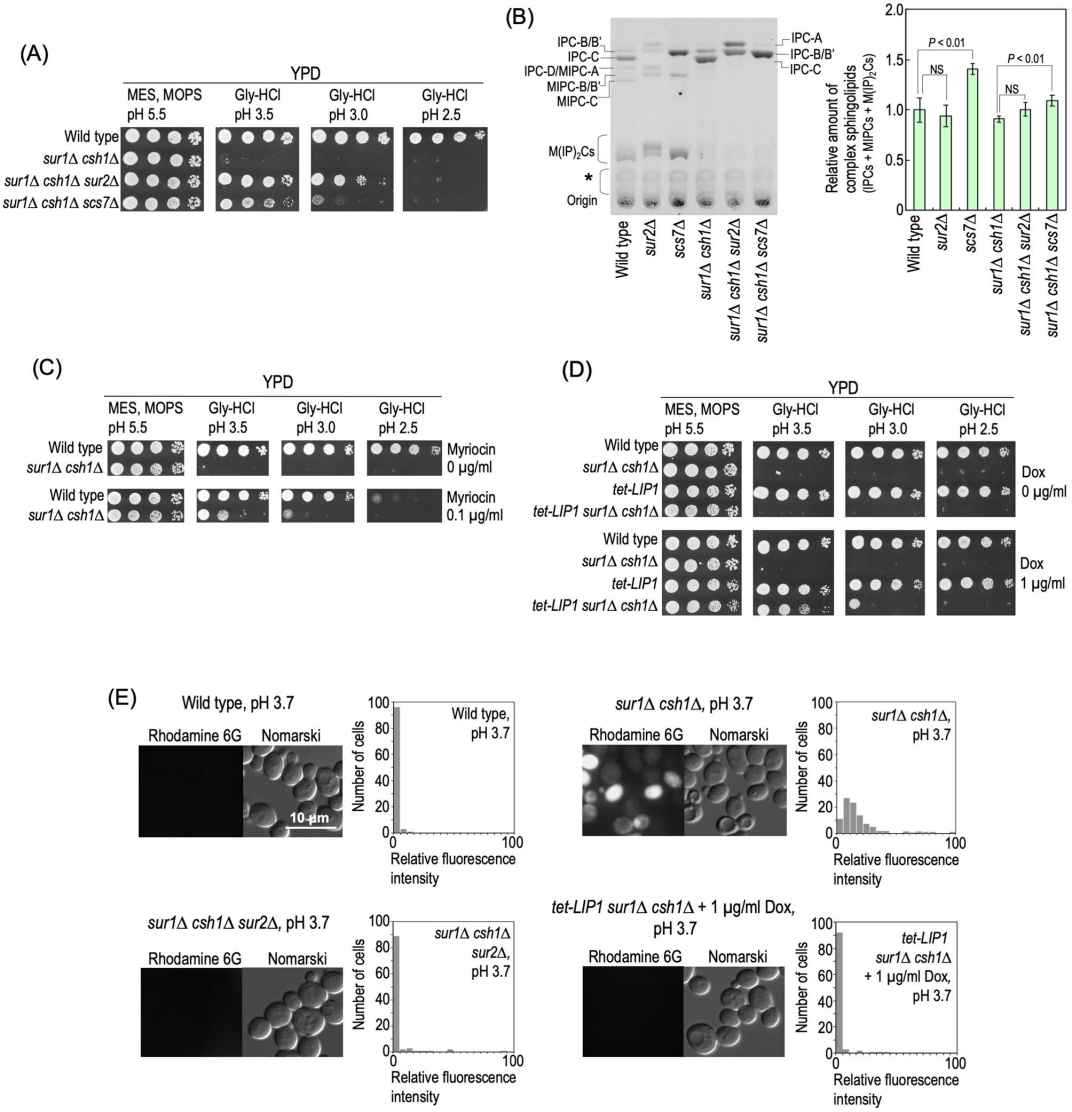
Repression of IPC biosynthesis confers resistance to low pH conditions in *sur1∆ csh1∆* cells. (**A**) Effects of deletion of *SUR2* and *SCS7* on the low pH hypersensitivity of *sur1∆ csh1∆* cells. Cells cultured in YPD medium were spotted onto agar plates containing YPD medium buffered to the indicated pH, and then incubated at 30 °C for 2 days. (**B**) TLC analysis of complex sphingolipids. Cells were cultured overnight in YPD medium, diluted (0.3 *A*_600_ units/ml) in fresh YPD medium, and then incubated for 5 h at 30 °C. Complex sphingolipids were analysed by TLC as described under METHODS. The amount of complex sphingolipids (IPCs, MIPCs, and M(IP)_2_Cs) in wild-type cells was taken as 1. Data represent means ± SD for one experiment (triplicate) representative of three independent experiments. NS, no significant difference. (**C**) Wild-type and *sur1∆ csh1∆* cells cultured in YPD medium were spotted onto agar plates containing YPD medium buffered to the indicated pH with or without 0.1 µg/ml myriocin, and then incubated at 30 °C for 2 days. (**D**) Wild-type, *sur1∆ csh1∆*, *tet-LIP1*, and *tet-LIP1 sur1∆ csh1∆* cells cultured in YPD medium with or without 1 µg/ml Dox were spotted onto agar plates containing YPD medium buffered to the indicated pH with or without 1 µg/ml Dox, and then incubated at 30 °C for 2 days. (**E**) Efficiency of incorporation of rhodamine 6G into cells. Incorporation efficiency of rhodamine 6G was examined as described in Fig. 3B. The details are given under METHODS.

Hypersensitivity to Ca^2+^, one of the typical phenotypes of MIPC biosynthesis-deficient mutants, is suppressed by several mutations (*lcb1*, *lcb2*, *tsc3*, *tsc10*, *tsc13*, *sur2*, and *scs7*), which suppress the accumulation of IPC-C due to a defect of MIPC biosynthesis^15^ (Figs. 1A, S2). Since *SUR2* involved in the generation of IPC-C through hydroxylation of the LCB moiety (Figs. 1A, S2) was found in our transposon mutagenesis screening (Fig. 4A), it was assumed that the low pH hypersensitivity of *sur1∆ csh1∆* cells is also caused by accumulation of IPC-C. Deletion of *SCS7* involved in generation of IPC-C (Figs. 1A, S2) also suppressed the low pH hypersensitivity of *SUR1*- and *CSH1*-deleted cells (Fig. 5A). In both wild-type and *sur1∆ csh1∆* backgrounds, the deletion of *SUR2* did not affect the total levels of complex sphingolipids; however, the deletion of *SCS7* caused a 1.4- to 1.2-fold increase in the total complex sphingolipid level (Fig. 5B). Deletion of combinations of *SUR1*, *CSH1*, *SUR2*, and *SCS7* did not cause significant changes in the levels of other membrane lipids, glycerophospholipids and ergosterol (Supplementary Fig. S3). Although the deletion of *SUR2* or *SCS7* caused the disappearance of IPC-C (Fig. 5B)^35^, *sur1∆ csh1∆ sur2∆* and *sur1∆ csh1∆ scs7∆* cells did not grow at pH 2.5 (Fig. 5A), suggesting that accumulation of IPCs other IPC-C also causes the low pH hypersensitivity. Treatment with myriocin, an inhibitor of serine palmitoyltransferase (SPT), causes reductions in the levels of all sphingolipids including IPCs. A suppressive effect on the hypersensitivity was also observed in the presence of a low concentration (0.1 µg/ml) of myriocin (Fig. 5C). To investigate whether or not repression of Cer synthase activity suppresses the low pH hypersensitivity of *sur1∆ csh1∆* cells, expression of *LIP1* encoding the regulatory subunit of Cer synthase^36^ was repressed. A mutant strain that carries the *LIP1* gene under the control of a tetracycline-regulatable promoter (*tet-LIP1*)^37,38^ was used, and doxycycline (Dox) was used for repression of expression by the tetracycline-regulatable promoter. Repression of expression of *LIP1* also improved the growth of *SUR1*- and *CSH1*-deleted cells at pH 3.5 and 3.0 (Dox-treated *sur1∆ csh1∆* versus Dox-treated *tet-LIP1 sur1∆ csh1∆* cells) (Fig. 5D). The increase in the plasma membrane permeability of *SUR1*- and *CSH1*-deleted cells at pH 3.7 was suppressed by the repression of *LIP1* or deletion of *SUR2* (*sur1∆ csh1∆* versus Dox-treated *tet-LIP1 sur1∆ csh1∆* or *sur1∆ csh1∆ sur2∆* cells) (Fig. 5E). Collectively, these results suggested that the low pH hypersensitivity of *sur1∆ csh1∆* cells is caused by accumulation of IPCs, especially IPC-C.

### Decreases in IPC levels under low pH conditions

**Figure 6:**
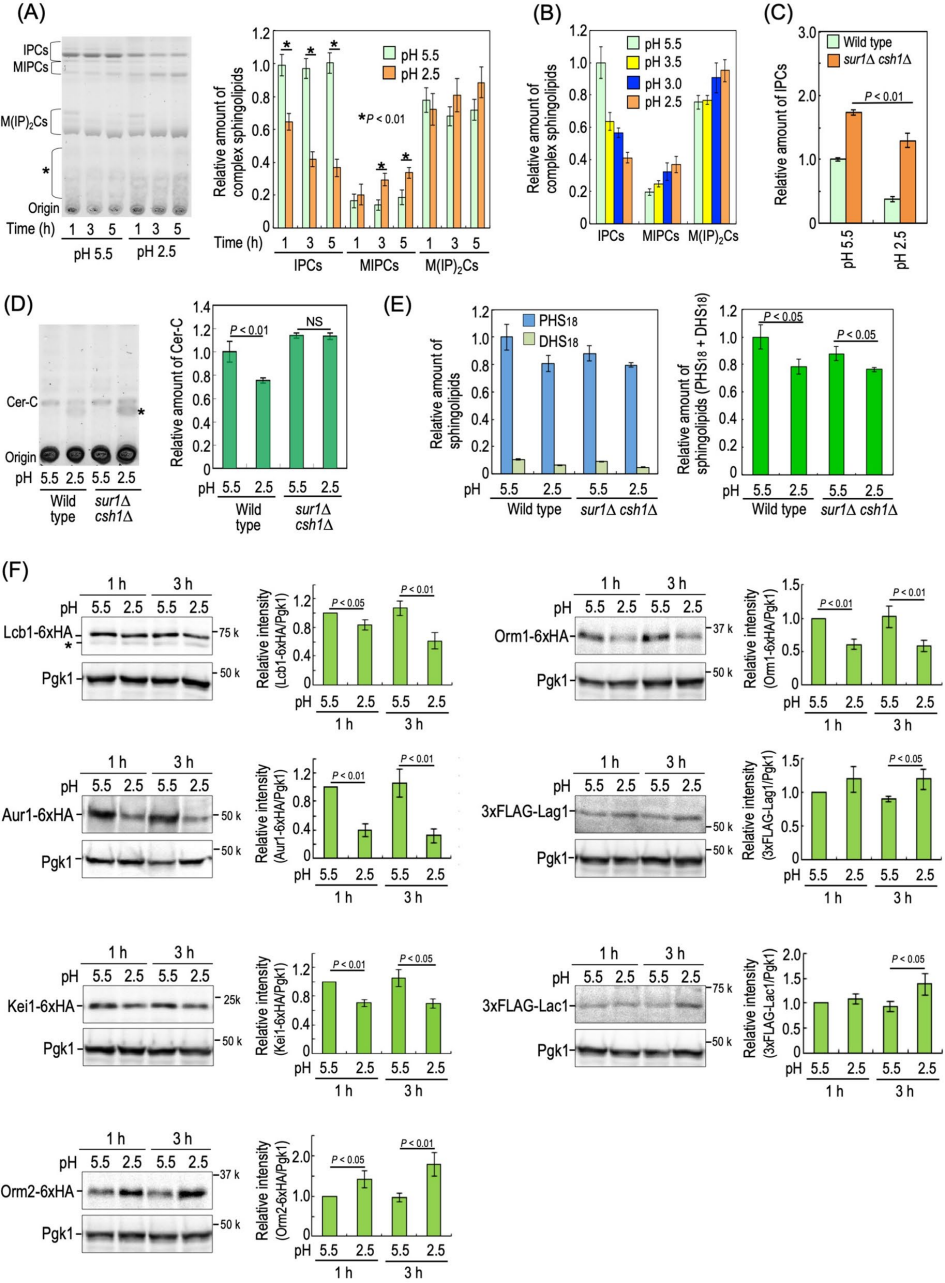
Analyses of sphingolipids and proteins involved in sphingolipid biosynthesis. (**A**) Wild-type cells were cultured overnight in YPD medium, diluted (0.7 *A*_600_ units/ml) in fresh YPD medium, and then incubated for 3.5 h at 30 °C. The cells were resuspended in fresh YPD medium buffered to pH 5.5 or 2.5 to 0.5 *A*_600_ units/ml, and then incubated for the indicated times at 30 °C. Complex sphingolipids were analysed by TLC. The amount of IPCs in wild-type cells incubated for 1 h at pH 5.5 was taken as 1. (**B**) Wild-type cells (0.5 *A*_600_ units/ml) were incubated in YPD medium buffered to the indicated pH for 3 h at 30 °C as described in Fig. 6A. Complex sphingolipids were analysed by TLC. The amount of IPCs in wild-type cells incubated at pH 5.5 was taken as 1. (**C** and **D**) Wild-type and *sur1∆ csh1∆* cells (0.5 *A*_600_ units/ml) were incubated in YPD medium buffered to pH 5.5 or 2.5 for 3 h at 30 °C as described in Fig. 6A. Lipids were analysed by TLC. The amount of IPCs (**C**) or Cer-C (**D**) in wild-type cells at pH 5.5 was taken as 1. The asterisks indicate unidentified bands. (**E**) Wild-type and *sur1∆ csh1∆* cells (0.5 *A*_600_ units/ml) were incubated in YPD medium buffered to pH 5.5 or 2.5 for 3 h at 30 °C as described in (**A**). Sphingolipids were hydrolysed with methanol/HCl, and then analyzed by reversed-phase HPLC. The values are the total sphingolipids containing PHS_18_ and/or DHS_18_. Data represent means ± SD for one experiment (triplicate) representative of three independent experiments. NS, no significant difference. (**F**) Cells (0.5 *A*_600_ units/ml) expressing each tagged protein were incubated in YPD medium buffered to pH 5.5 or 2.5 for the indicated times at 30 °C as described in (**A**). Yeast cell extracts were immunoblotted using anti-HA, anti-FLAG or anti-Pgk1 antibodies. The amount of Lcb1-6xHA, Aur1-6xHA, Kei1-6xHA, Orm2-6xHA, Orm1-6xHA, 3xFLAG-Lag1, or 3xFLAG-Lac1/Pgk1 in cells incubated for 1 h at pH 5.5 was taken as 1. Data represent means ± SD for three independent experiments. Full Western blots are shown in Supplementary Fig. S10, *panels c–i*. The details are given under METHODS.

Since mutations causing accumulation of IPCs confer low pH hypersensitivity, we next investigated whether or not a change in the level of IPCs in wild-type cells is observed under low pH conditions. As shown in Fig. 6A, the IPC levels in wild-type cells began to decrease when cells were exposed to pH 2.5 medium for 1 h, and after 5-h incubation at pH 2.5, an approximately 60% decrease in the IPC levels was observed as compared with incubation at pH 5.5. Conversely, the MIPC levels increased approximately twofold at pH 2.5 (Fig. 6A). A change in the complex sphingolipid levels was similarly observed when the low pH culture medium was prepared by different means other than with glycine–HCl (Supplementary Fig. S4). A decrease in the IPC levels was also observed at pH 3.5 and 3.0; however, the most notable effect was observed at pH 2.5 (Fig. 6B). Decrease in the IPC levels at pH 2.5 was also observed in *sur1∆ csh1∆* cells; however, the IPC levels in *sur1∆ csh1∆* cells at pH 2.5 were much higher than those in wild-type cells (Fig. 6C). The level of Cer-C (the most major type of Cer in yeast^35,39^) in wild-type cells incubated at pH 2.5 decreased to approximately 75% of that at pH 5.5 (Fig. 6D). In contrast, such a decrease was not observed in *sur1∆ csh1∆* cells (Fig. 6D). Figure 6E shows the results of quantification of the levels of all sphingolipids containing PHS_18_ and DHS_18_^40^. In both wild-type and *sur1∆ csh1∆* cells, significant decreases in the PHS_18_- and DHS_18_-based sphingolipid levels were observed under the pH 2.5 conditions (Fig. 6E). On the other hand, wild-type cells did not exhibit significant changes in the levels of other membrane lipids, glycerophospholipids and ergosterol, under the pH 2.5 conditions (Supplementary Fig. S5).

Next, we examined the expression levels of Lcb1 (catalytic subunit of SPT)^41^, Aur1 (IPC synthase)^42^, Kei1 (essential component of IPC synthase)^43^, Orm1 and Orm2 (negative regulators of SPT)^44^, and Lag1 and Lac1 (Cer synthases)^45^, all of which are involved in regulation of the IPC and Cer levels in cells. To detect the expression of these proteins, the chromosomal genes were tagged with 6xHA at the C-terminus or 3xFLAG at the N-terminus in wild-type cells^22,40^ (The band of each protein tagged with 6xHA or 3xFLAG was not detected for untagged cells (Supplementary Fig. S6)). The expression levels of Lcb1, Aur1, and Kei1 decreased to approximately 60, 30, and 70%, respectively, in cells incubated at pH 2.5 compared to pH 5.5 (Fig. 6F), which may explain the decreases in the levels of sphingolipids including IPCs under the low pH conditions. In addition, the Orm2 expression level in cells incubated at pH 2.5 for 3 h was significantly increased as compared with that at pH 5.5 (Fig. 6F). In contrast, the Orm1 expression level decreased under low pH conditions (Fig. 6F). The inhibitory activity of Orm2 and Orm1 toward SPT is downregulated by their phosphorylation^44^, and thus we also examined the phosphorylation states of Orm2 and Orm1 in cells cultured at pH 2.5 and pH 5.5. The degree of phosphorylation was determined by phos-tag SDS-PAGE^39^. As shown in Supplementary Fig. S7, the phosphorylated form of Orm2 did not increase at pH 2.5, suggesting that the Orm2 protein that increased under low pH conditions is an active form inhibiting SPT activity. Both the phosphorylated and dephosphorylated forms of Orm1 decreased under low pH conditions (Supplementary Fig. S7). The expression levels of Lag1 and Lac1 were slightly but significantly increased at pH 2.5 (Fig. 6F); however, the physiological significance of these changes remains unknown.

### Simultaneous upregulation of IPC synthase and SPT causes hypersensitivity to low pH conditions

**Figure 7:**
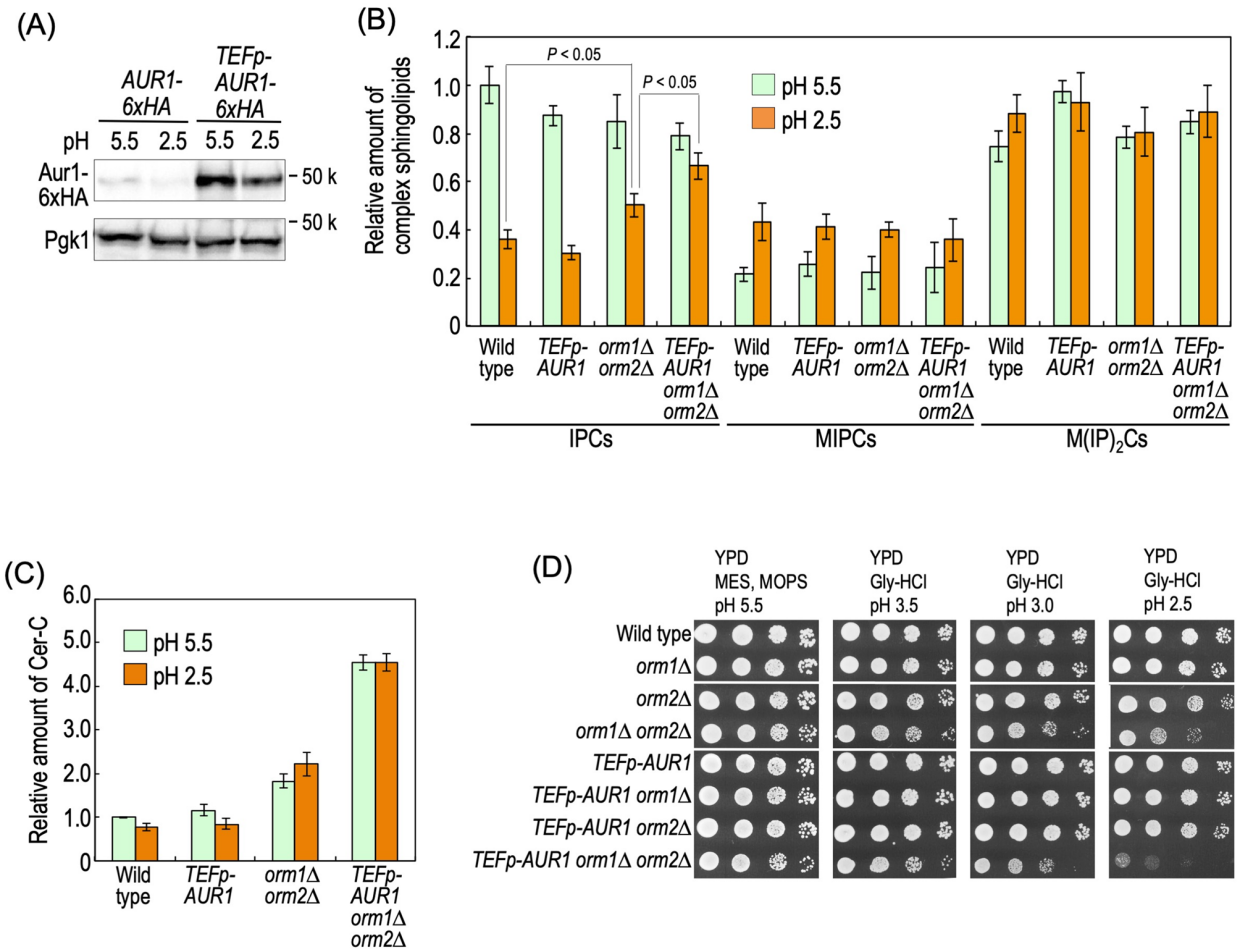
Effects of overexpression of *AUR1,* and deletion of *ORM1* and *ORM2* on sphingolipid levels and sensitivity to low pH conditions. (**A**) Western blotting analysis of overexpressed Aur1-6xHA due to the TEF promoter. Cells expressing Aur1-6xHA with the native promoter or TEF promoter (0.5 *A*_600_ units/ml) were incubated in YPD medium buffered to pH 5.5 or 2.5 for 3 h at 30 °C as described in Fig. 6A. Yeast cell extracts were immunoblotted using anti-HA or anti-Pgk1. Full Western blots are shown in Supplementary Fig. S10, *panel j*. (**B** and **C**) Effects of *TEFp-AUR1* and/or *orm1∆ orm2∆* on the complex sphingolipid (**B**) and Cer-C (**C**) levels. Wild-type, *TEFp-AUR1*, *orm1∆ orm2∆,* and *TEFp-AUR1 orm1∆ orm2∆* cells (0.5 *A*_600_ units/ml) were incubated in YPD medium buffered to the indicated pH for 3 h at 30 °C as described in Fig. 6A. Lipids were extracted and analyzed as described under METHODS. The amount of IPCs (**B**) or Cer-C (**C**) in wild-type cells at pH 5.5 was taken as 1. Data represent means ± SD for one experiment (triplicate) representative of three independent experiments. (**D**) Cells cultured in YPD medium were spotted onto agar plates containing YPD medium buffered to the indicated pH, and then incubated at 30 °C for 2 days. The details are given under METHODS.

Since accumulation of IPCs causes low pH hypersensitivity, it is assumed that the decreases in IPC levels in cells incubated at low pH conditions (Fig. 6) are a protective response to extracellular low pH. Thus, we next examined the effects of forced upregulation of IPC synthase and SPT under low pH conditions. To do this, chromosomal *AUR1* was overexpressed under the control of a constitutive TEF promoter (*TEFp-AUR1*)^46^. The overexpression of Aur1 by the TEF promoter was confirmed by tagging with 6xHA at the C-terminus of Aur1 in cells cultured at both pH 5.5 and 2.5 (Fig. 7A). In addition, for upregulation of in vivo SPT activity, *ORM1/2* was deleted^44^. Deletion of *ORM1/2* caused slight but significant increases in the IPC levels in cells cultured at pH 2.5 (wild-type versus *orm1∆ orm2∆* cells at pH 2.5), and the overexpression of *AUR1* enhanced the increases in the IPC levels in *orm1∆ orm2∆* cells (*orm1∆ orm2∆* versus *TEFp-AUR1 orm1∆ orm2∆* cells at pH 2.5) (Fig. 7B). In contrast, *TEFp-AUR1* alone did not affect the IPC levels at pH 2.5. It should be noted that the increases in IPC levels on the deletion of *ORM1/2* and *TEFp-AUR1* were not observed when cells were cultured at pH 5.5 (Fig. 7B), suggesting that these mutations affect the IPC levels only when the IPC levels are downregulated under low pH conditions. This supports the notion that the decreases in IPC levels under low pH conditions are caused by downregulation of the in vivo activities of IPC synthase and SPT. The deletion of *ORM1/2* caused increases in the Cer-C levels in cells cultured at both pH 5.5 and 2.5, and the increases were greatly enhanced on the overexpression of Aur1 (Fig. 7C). Under low pH conditions, *orm1∆ orm2∆* cells exhibited slow growth as compared with wild-type cells, and a more severe growth defect was observed in *TEFp-AUR1 orm1∆ orm2∆* cells (Fig. 7D). Thus, collectively, it was suggested that the downregulation of IPC synthase and SPT is required for the maintenance of cell growth under low pH conditions.

### Change in ergosterol homeostasis at plasma membranes rescues the growth defect of *sur1∆ csh1∆* cells under low pH conditions

**Figure 8:**
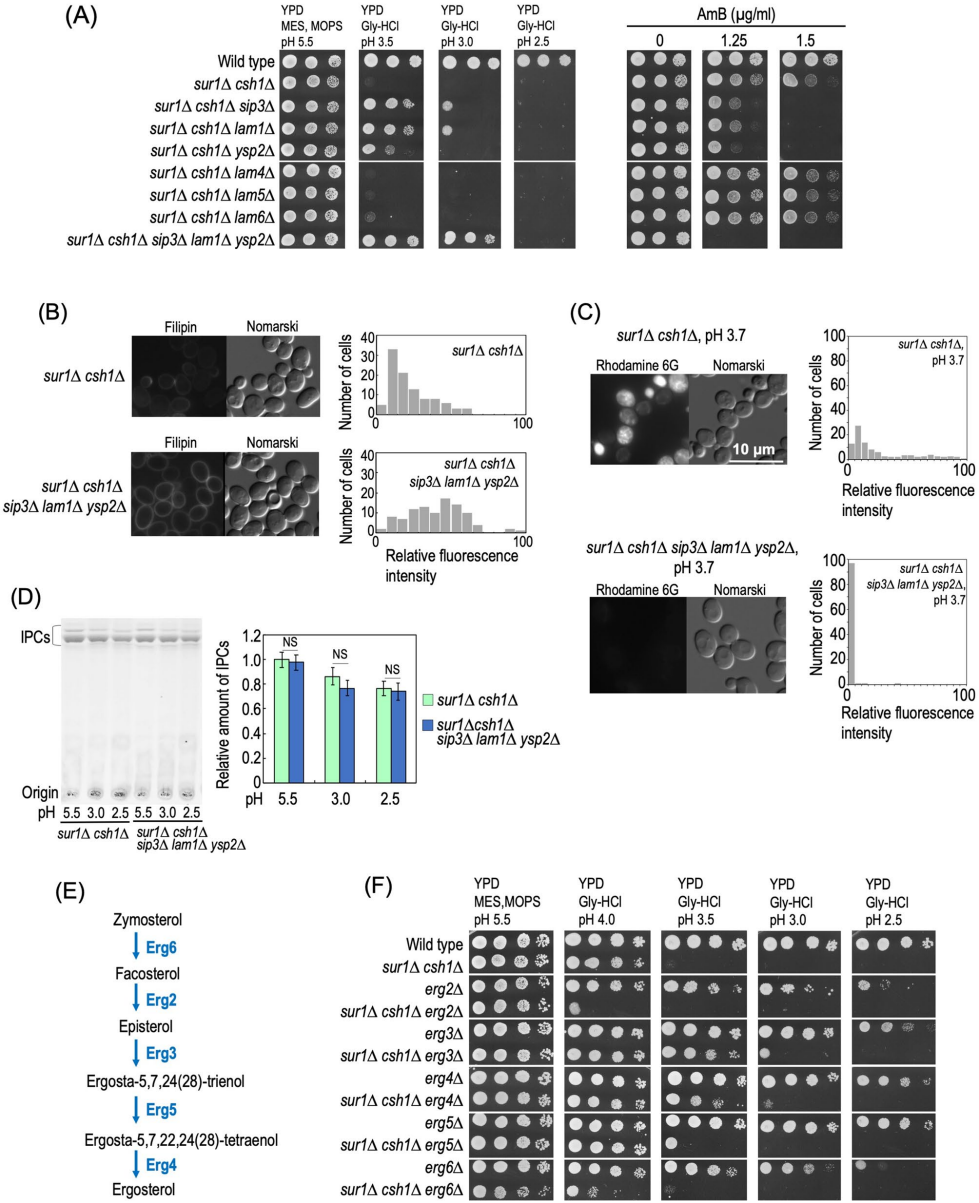
Involvement of ergosterol at plasma membranes in the low pH hypersensitivity of *sur1∆ csh1∆* cells. (**A**) Effects of deletion of genes encoding LAM family proteins on sensitivity to low pH conditions and amphotericin B (AmB) of *sur1∆ csh1∆* cells. Cells cultured in YPD medium were spotted onto agar plates containing YPD medium buffered to the indicated pH or YPD medium (pH 6.0) containing the indicated amounts of AmB, and then incubated at 30 °C for 2 days. (**B**) *sur1Δ csh1Δ* and *sur1Δ csh1Δ sip3∆ lam1∆ ysp2∆* cells grown to the exponential phase were stained with filipin. The graphs indicate the frequency distributions of filipin fluorescence intensity in individual cells. Data represent the values for 100 cells for individual strains. (**C**) Efficiency of incorporation of rhodamine 6G into *sur1Δ csh1Δ* and *sur1Δ csh1Δ sip3∆ lam1∆ ysp2∆* cells. Incorporation efficiency of rhodamine 6G was examined as described in Fig. 3B. (**D**) Effect of deletion of *SIP3*, *LAM1*, and *YSP2* on IPC levels of *SUR1*- and *CSH1*-deleted cells. TLC analysis of complex sphingolipids was performed as described in Fig. 6C. (**E**) The later stages of the ergosterol biosynthesis pathway in *S. cerevisiae*. Proteins responsible for the synthesis are shown. (**F**) Cells cultured in YPD medium were spotted onto agar plates containing YPD medium buffered to the indicated pH, and then incubated at 30 °C for 2 days. The details are given under METHODS.

*LAM1* and *SIP3*, which were found in the transposon mutagenesis screening (Fig. 4A), encode some of the lipid transfer proteins anchored at a membrane contact site (LAM) family, which includes Lam1, Sip3, Ysp2, Lam4, Lam5, and Lam6^47^. Lam1, Sip3, and Ysp2, which are localized membrane contact sites between the endoplasmic reticulum (ER) and plasma membranes, are involved in retrograde trafficking of sterols between the two membranes, and *lam1∆*, *sip3∆*, and *ysp2∆* cells exhibit hypersensitivity to the polyene antifungal amphotericin B (AmB), which exerts cytotoxicity by binding to ergosterol at plasma membranes, suggesting that these mutations change the distribution pattern of ergosterol at plasma membranes^47,48^ (Supplementary Fig. S8). In contrast, a lack of Lam5 or Lam6, which are localized to membrane contact sites other than plasma membranes, does not affect sensitivity to AmB^47,49,50^ (Supplementary Fig. S8). The deletion of *LAM1*, *SIP3*, or *YSP2,* but not *LAM4*, *LAM5,* or *LAM6,* in *SUR1-* and *CSH1*-deleted cells suppressed the hypersensitivity to low pH conditions and enhanced the sensitivity to AmB (Fig. 8A). Furthermore, simultaneous deletion of *LAM1*, *SIP3,* and *YSP2* resulted in the strongest effect on the sensitivity to low pH conditions and AmB in *SUR1-* and *CSH1-*deleted cells (*sur1∆ csh1∆ sip3∆ lam1∆ ysp2∆* cells (Fig. 8A)). In contrast, the triple deletion of *LAM1*, *SIP3,* and *YSP2* did not affect the cell growth at pH 2.5 in the wild-type background (Supplementary Fig. S8), indicating that the effectiveness of deletion of LAM family genes for the low pH sensitivity is only observed in MIPC biosynthesis-deficient cells. An increase in the cell-surface ergosterol level due to the triple deletion of *LAM1*, *SIP3,* and *YSP2* in the *sur1∆ csh1∆* background was observed when cells were stained with filipin, a fluorescent probe staining sterols (Fig. 8B)^51^. Notably, the increase in plasma membrane permeability in *sur1∆ csh1∆* cells incubated at pH 3.7 was greatly suppressed by the triple deletion of *LAM1*, *SIP3,* and *YSP2* (Fig. 8C), suggesting restoration of the impairment of plasma membrane integrity. The triple deletion of *LAM1*, *SIP3,* and *YSP2* did not cause changes in the IPC levels of *SUR1*- and *CSH1*-deleted cells incubated at pH 5.5, 3.5, or 2.5, indicating that the suppressive effect of a defect of LAM family proteins is not due to regulation of complex sphingolipid biosynthesis (Fig. 8D). Collectively, these results suggested that the change in ergosterol homeostasis at plasma membranes confers robustness to *sur1∆ csh1∆* cells under low pH conditions.

To gain further insight into the relationship between complex sphingolipids and ergosterol under low pH conditions, we used mutants as to the ergosterol biosynthesis pathway (Fig. 8E,F). Deletion of *ERG6*, *ERG2*, *ERG3*, *ERG5* or *ERG4*, which are involved in the final stages of the ergosterol biosynthesis pathway (Fig. 8E), does not cause a lethal phenotype; however, sterol intermediates that can partly but not completely substitute for the biological roles of ergosterol are accumulated instead of ergosterol in such mutant cells^52^. Deletion of *ERG2* or *ERG6* enhanced the low pH hypersensitivity of *SUR1*- and *CSH1*-deleted cells (*sur1∆ csh1∆* versus *sur1∆ csh1∆ erg2∆* or *sur1∆ csh1∆ erg6∆* cells) (Fig. 8F). In contrast, deletion of *ERG3*, *ERG4*, or *ERG5* suppressed the low pH hypersensitivity of *SUR1*- and *CSH1*-deleted cells (*sur1∆ csh1∆* versus *sur1∆ csh1∆ erg3∆*, *sur1∆ csh1∆ erg4∆,* or *sur1∆ csh1∆ erg5∆* cells) (Fig. 8F). These results indicated that the low pH hypersensitivity of *sur1∆ csh1∆* cells is affected by changes in the detailed structure of ergosterol.

## Discussion

**Figure 9:**
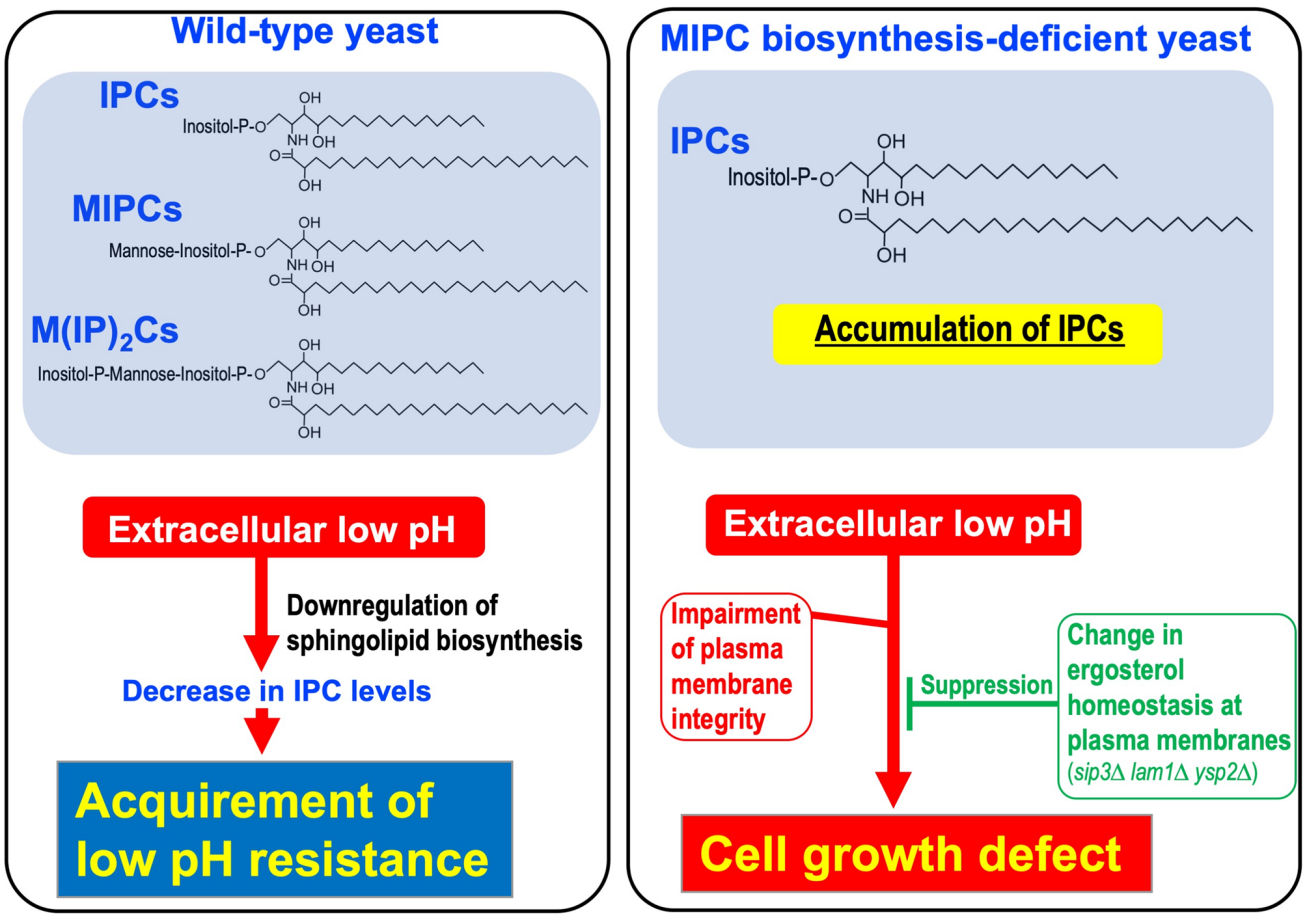
Relationship IPC levels and sensitivity to low pH conditions. When wild-type cells are exposed to low pH, cellular IPC levels immediately decrease, which is required for acquirement of resistance to the low pH conditions. In contrast, in *sur1∆ csh1∆* cells, IPCs are highly accumulated due to the loss of MIPC biosynthesis, and thus a strong growth defect is induced under low pH conditions. However, the low pH hypersensitivity of *sur1∆ csh1∆* cells is suppressed by a change in ergosterol homeostasis at plasma membranes, suggesting the functional relationship between complex sphingolipids and ergosterol under low pH conditions.

In this study, it was found that accumulation of IPCs due to a defect of MIPC biosynthesis causes hypersensitivity to low pH culture conditions. Importantly, decreases in IPC levels were observed when wild-type yeast cells were incubated under low pH conditions (Fig. 6A,B), and enhancement of the biosynthesis of IPCs conferred hypersensitivity to low pH conditions (Fig. 7). Thus, it was suggested that yeast cells protect themselves against extracellular low pH through downregulation of IPC levels (Fig. 9). Expression of protein levels involved in IPC biosynthesis changed under low pH conditions (Fig. 6F), which may contribute to the decreases in the levels of sphingolipids including IPCs under low pH conditions. At present, it remains unclear how these protein levels are regulated under low pH conditions. It was reported that the Orm2 expression level is regulated by calcineurin and the calcineurin-activated transcription factor^39,53^, and degradation of Orm2 is controlled through the endosome and Golgi-associated degradation pathway (EGAD)^54^. It should be noted that *PMR1* encoding P-type Ca^2+^/Mn^2+^-ATPase mainly located in the Golgi apparatus, which was found in our transposon mutagenesis screening (Fig. 4A), affects activation of calcineurin through regulation of cytosolic Ca^2+^^55^. In addition, reduction in IPC levels through a reduced transcriptional level of *AUR1* was reported in cells lacking *PHO85* encoding cyclin-dependent kinase involved in the phosphate-sensing signaling pathway^56^. Thus, involvement of these signaling pathways in changes in the expression levels of Orm2 and Aur1 under low pH conditions should be addressed in the future. On the other hand, in the acquirement of resistance to low pH conditions, involvement of a homeostatic regulation system for sphingolipid biosynthesis, such as the TORC2-Ypk1 pathway^57^, should also be considered. To examine whether or not Ypk1/2, protein kinases having central roles in upregulation of sphingolipid biosynthesis, are related to the acquirement of resistance to low pH conditions, temperature-sensitive mutant cells of *YPK1* (*ypk1-ts ypk2Δ* cells)^58^ were used (Supplementary Fig. S9). Mutations of *YPK1* and *YPK2* cells did not improve the cell growth under low pH conditions (Supplementary Fig. S9). This may suggest that regulation of the TORC2-Ypk1 pathway does not positively contribute to the acquirement of low pH resistance.

The expression level of Orm2 increased under low pH conditions, whereas, conversely, that of Orm1 decreased (Fig. 6F). Although Orm1 and Orm2 have redundant functions in negative regulation of de novo sphingolipid biosynthesis, the contribution of Orm1 is smaller than that of Orm2; that is, the single deletion of *ORM2* but not *ORM1* causes increases in the total sphingolipid and LCB levels^40,59,60^. Thus, it is likely that decreases in the levels of sphingolipids including IPCs under low pH conditions are preferentially relevant to the increase in the Orm2 level. On the other hand, it was reported that activation of Orm1/2 by Npr1 kinase stimulates complex sphingolipid biosynthesis, which is not mediated through interaction between Orm1/2 and SPT^61^. However, it is likely that this effect of Orm1/2 is cancelled under low pH conditions because the expression level of Aur1 is markedly decreased at pH 2.5 (Fig. 6F).

Under low pH conditions, wild-type yeast cells exhibited not only decreases in IPC levels, but also a slight decrease in the Cer-C level and increases in MIPC levels (Fig. 6A,B,D). Since deletion of *SUR1* and *CSH1* causes loss of MIPCs, and the decrease in the Cer-C level under low pH conditions was not observed in *sur1∆ csh1∆* cells (Fig. 6C,D), the possibility that the changes in the levels of Cers and MIPCs are also related to the acquirement of resistance to low pH conditions should be considered. At pH 2.5, *TEFp-AUR1 orm1∆ orm2∆* cells, but not *sur1∆ csh1∆* cells, exhibited an around fivefold increase in the Cer-C level as compared with wild-type cells; however, the low pH hypersensitivity of *TEFp-AUR1 orm1∆ orm2∆* cells was less severe than that of *sur1∆ csh1∆* cells (Fig. 7D versus Fig. 1C). Moreover, at pH 2.5, the increases in the IPC levels in *TEFp-AUR1 orm1∆ orm2∆* cells were lower than those in *sur1∆ csh1∆* cells (Fig. 7B versus Fig. 6C). Thus, it is indicated that accumulation of IPCs is much more detrimental than that of Cers under low pH conditions. In addition, the MIPC levels were not affected by the overexpression of *AUR1* and/or the deletion of *ORM1* and *ORM2* (Fig. 7B), which suggests that the changes in MIPC levels at pH 2.5 are not related with low pH sensitivity.

In this study, we found the relationship between complex sphingolipids and ergosterol in the maintenance of cell growth under low pH conditions; that is, deletion of *LAM1*, *SIP3*, and *YSP2* encoding proteins involved in sterol transfer between the ER and plasma membranes improved the growth defect, and suppressed the plasma membrane permeability of *SUR1*- and *CSH1*-deleted cells under low pH conditions (Fig. 8A,C). Furthermore, deletion of *ERG2* or *ERG6* enhanced the low pH hypersensitivity of *SUR1*- and *CSH1*-deleted cells (Fig. 8F). *ERG6* encodes sterol C-24 methyltransferase, an enzyme involved in methylation at position 24 of the side chain of the sterol backbone. It was proposed that hydrophobic interaction between the C24-methyl group and fatty acid moiety of sphingolipids is important for the formation of microdomain rafts^62^. Thus, it is possible that the decrease in the interaction between complex sphingolipids and sterols caused by the removal of the methyl group at position 24 due to the deletion of *ERG6* increases the sensitivity to low pH in *SUR1*- and *CSH1*-deleted cells. Furthermore, the deletion of *ERG6* causes increase in plasma-membrane permeability^33^, which may be related to the enhancement of low pH sensitivity. Among *erg* mutants (*erg2∆*, *erg3∆*, *erg4∆*, *erg5∆,* and *erg6∆* cells), deletion of *ERG6* or *ERG2* confers strong resistance to nystatin that binds to ergosterol within plasma membranes and exerts cytotoxicity^52,63^, which may suggest that the nystatin-accessible sterol content at plasma membranes is decreased on the deletion of *ERG6* or *ERG2.* Thus, it is also possible that a change in the distribution pattern of sterols at plasma membranes on the deletion of *ERG6* or *ERG2* affects the pH sensitivity of *SUR1*- and *CSH1*-deleted cells. This is consistent with the fact that the deletion of *LAM1*, *SIP3*, and *YSP2* that causes hypersensitivity to AmB confers the resistance to low pH conditions in *SUR1*- and *CSH1*-deleted cells (Fig. 8A). It should be noted that the effects of deletion of *ERG* genes on the pH sensitivity of *sur1∆ csh1∆* cells are somewhat different from those on the cell wall integrity defect of *sur1∆ csh1∆* cells^51^. For example, the deletion of *ERG4* has facilitatory effects on SDS and caffeine hypersensitivities, which are hall mark features of a defect in cell wall integrity^51^; however, the deletion had the opposite effect on the low pH sensitivity (Fig. 8F). This is probably because the causative factor leading to low pH hypersensitivity in MIPC biosynthesis-deficient cells is different from that leading to the cell wall integrity defect in the cells; that is, the low pH hypersensitivity is caused by the accumulation of IPCs whereas the cell wall integrity defect is caused by loss of MIPCs^16^.

Several groups indicated that ergosterol plays important roles in maintenance of the rigidity of plasma membranes including maintenance of membrane permeability and fluidity^33,52,64^. Therefore, the genetic interactions between MIPC synthase and sterol-related genes suggest that a defect of MIPC biosynthesis affects the physical properties of plasma membranes under low pH conditions. We showed that *sur1∆ csh1∆* cells exhibit increased plasma membrane permeability under low pH conditions (Fig. 3B). At present, it remains unclear why plasma membrane permeability in *sur1∆ csh1∆* cells is increased only when the culture medium is acidic. In silico membrane simulations suggested that complex sphingolipids increases in membrane thickness and decreases the lipid bilayer permeability of extracellular acetic acid^9^. Moreover, in *Z. bailii*, myriocin treatment causes increased incorporation of acetic acid into cells^9^. We also confirmed that, at pH 4.0, intracellular acidification by acetic acid was enhanced by the deletion of *SUR1* and *CSH1* (Fig. 2E). These results support the notion that complex sphingolipids play important roles in maintenance of plasma-membrane permeability. It should be noted that the subcellular distribution patterns of IPCs, MIPCs, and M(IP)_2_Cs are different; that is, MIPCs and M(IP)_2_Cs are especially enriched in plasma membranes, whereas IPCs are widely distributed in plasma membranes, vacuoles, and the Golgi^65^. Thus, it is possible that the deletion of *SUR1* and *CSH1* affects the plasma membrane distribution of complex sphingolipids, and consequently decreases plasma-membrane permeability under low pH conditions.

Uemura et al. reported that, under neutral pH conditions, the lateral diffusion speed of enhanced GFP (EGFP)-tagged Hxt1, plasma membrane-localized hexose transporter 1, is decreased in *sur1∆ csh1∆ sur2∆* and *sur1∆ csh1∆ sur2∆ scs7∆* cells, but not in *sur1∆ csh1∆*, or *scs7∆* cells^8^. This phenomenon seems not to be directly related to our results because the low pH hypersensitivity of *sur1∆ csh1∆* cells is suppressed by the deletion of *SUR2* or *SCS7* (Fig. 5A). In addition, the distributions of EGFP-tagged Tat1, Can1, and Pma1, typical plasma membrane-localized proteins, in wild-type and *sur1∆ csh1∆* cells did not clearly differ even at low pH conditions (Tani M, unpublished results). However, further detailed evaluation of various plasma membrane properties of *sur1∆ csh1∆* cells cultured under low pH conditions is required in the future.

Besides *SUR2*, *LAM1*, *SIP3,* and *PMR1*, *LCB4* and *XRN1* were also found in the transposon mutagenesis screening (Fig. 4A). *LCB4* encodes LCB kinase, which converts LCBs to LCB 1-phosphates^3^. This phosphorylation is essential for catabolism of LCBs to phosphoethanolamine and acyl-CoAs, both of which are precursors of membrane phospholipids^66^. We also investigated the effects of deletion of *DPL1* and *HFD1* encoding LCB 1-phosphates lyase and fatty aldehyde dehydrogenase, respectively, both of which are involved in the catabolism of LCBs^3,66^, and found that these deletions also confer resistance to low pH conditions in *sur1∆ csh1∆* cells (Otsu M and Tani M, unpublished results). Thus, some relevance between the LCB catabolic pathway and the low pH sensitivity was suggested. Recently, it was reported that Xrn1, a 5′-3′ exonulease involved in mRNA degradation in the cytosol, regulates translation of a specific group of transcripts encoding membrane proteins^67^. Thus, dysregulation of the expression of some membrane protein(s) due to the deletion of *XRN1* may confer resistance to low pH conditions in *sur1∆ csh1∆* cells.

In summary, the present study indicated the importance of proper regulation of the IPC levels for acquirement of resistance to low extracellular pH. Further detailed analyses of this molecular mechanism will provide a novel insight into the relationship between regulation of complex sphingolipids and environmental stress responses.

## Methods

### Yeast strains and media

The *S. cerevisiae* strains used are listed in Supplementary Table S2. Disruption of genes was performed by replacing their open reading frames with the *kanMX4* marker from a genome from a yeast knockout library or the pFA6a-kanMX4 vector, the *hphNT1* marker from the pFA6a-hphNT1 vector^46,68^, the *natMX4* marker from the p4339 vector (pCRII-TOPO::natMX4)^69^, the *URA3* marker from pRS406, or the *LEU2* marker from the pRS405 vector^70^. For overexpression of genes by a strong and constitutive TEF promoter, a TEF promoter cassette containing the *natNT2* marker from pYM-N19 was introduced immediately upstream of the initiator ATG of the chromosomal gene, as described previously^46^. To generate cells expressing *PMA1* under the control of ADH promoter, an ADH promoter cassette containing the *kanMX4* marker from pYM-N18 was introduced immediately upstream of the initiator ATG of chromosomal *PMA1*, as described previously^46^. To tag the N-terminus of Lag1 or Lac1 with three copies of the FLAG epitope (3xFLAG), a 3xFLAG tag was introduced immediately downstream of the initiator ATG of chromosomal *LAG1* or *LAC1* without changing the potential promoter region according to the method described previously^71^. A DNA fragment of the *LAG1* or *LAC1* ORF without the initiator ATG was amplified by PCR using LAG1-3xFLAG-HindIII-F and LAG1-3xFLAG-BamHI-R (for *LAG1*), or LAC1-3xFLAG-HindIII-F and LAC1-3xFLAG-BamHI-R (for *LAC1*), and yeast genomic DNA as a template (The sequences of the oligonucleotide primers used are listed in Supplementary Table S3). The PCR products were inserted into the *Hind*III and *BamH*I sites of the p3xFLAG-CMV-7.1 vector (Sigma-Aldrich, St. Louis, MO, USA). A DNA fragment of *3xFLAG-LAG1* or *3xFLAG-LAC1* was amplified by PCR using 3xFLAG-LAG1-F1 and LAG1Hyg-R (for *LAG1*) or 3xFLAG-LAC1-F1 and LAC1Hyg-R (for *LAC1*), and the p3xFLAG-CMV-7.1 vector containing the *LAG1* or *LAC1* ORF as a template. A DNA fragment containing the *hphNTI* marker was amplified by PCR using LAG1Hyg-F and LAG1-S2 (for *LAG1*) or LAC1Hyg-F and LAC1-S2 (for *LAC1*), and pYM16^46^ as a template. These two DNA fragments were combined by PCR, and the resultant DNA was used to transform cells. The cells were cultured in YPD medium (1% yeast extract, 2% peptone, and 2% glucose (pH 6.0)), or SC (synthetic complete) medium (0.67% yeast nitrogen base without amino acids (BD Difco, Heidelberg, Germany) and 2% glucose) containing nutritional supplements. Buffered medium was prepared by the addition of 50 mM MES and 50 mM MOPS (for pH 5.5), 100 mM Gly-HCl (for pH 4.0, 3.7, 3.5, 3.0, and 2.5), and 100 mM phospholic acid-sodium dihydrogen phosphate (for pH 2.5). Medium adjusted to pH 2.5 was also prepared by the addition of HCl.

### Plasmids

A single-copy plasmid (pRS416) containing *SUR1-6xHA,* and its 5′- and 3′-untranslated regions (500 and 74 bp, respectively) was constructed as described below. A DNA fragment containing *SUR1-6xHA* was amplified by PCR using SUR1-6HA-SacI-S and SUR1-6HA-KpnI-A, and genomic DNA of the BY4741 strain expressing *SUR1-6xHA* (MTY1315)^22^ as a template. The fragment obtained was subcloned into pRS416. The DNA sequence was verified with an ABI PRISM 3,100 genetic analyzer (Applied Biosystems, Foster, CA). pKL06, a plasmid expressing super ecliptic pHluorin-mRuby2 fusion protein, was obtained from Addgene (plasmid 104,430).

### Spot assay

Cells were cultured overnight in YPD or SC-Ura medium at 30 °C, and then spotted onto agar plates containing YPD medium buffered to pH 5.5, 4.0, 3.5, 3.0, or 2.5 in tenfold serial dilutions starting with a density of 0.7 *A*_600_ units/ml. All plates were incubated at 30 °C and photographed after 1 or 2 days.

### Cell viability

Cells were cultured overnight in YPD medium, diluted (1 *A*_600_ units/ml) in fresh YPD medium, and then incubated for 3 h at 30 °C. The cells were resuspended in fresh YPD medium buffered to pH 5.5, 3.7, or 2.5 to 0.7 *A*_600_ units/ml, and then incubated for 1, 2, 3, or 5 h at 30 °C. Then equal numbers of cells, as determined from *A*_600_, were plated onto YPD plates, and incubated for 2 days at 30 °C, and then the numbers of colonies on the plates were determined. Relative colony forming units (CFU) were calculated as follows: relative CFU (%) = (colony numbers of each sample/colony numbers of wild-type cells incubated at pH 5.5) × 100.

### Transposon mutagenesis screening

Mutagenesis by random insertion of the transposon mTn-*lacZ/LEU2* was performed as described previously^34^ using a yeast genomic library kindly provided by Dr. Michael Snyder (Yale University, New Haven, CT) and Dr. Akio Kihara (Hokkaido University, Sapporo, Japan). The library was digested with *Not*I, and the resultant DNA fragments were transformed into *sur1∆ csh1∆* cells. The transformed cells were plated on SC plates lacking leucine buffered to pH 3.7, on which a strong growth defect of *sur1∆ csh1∆* cells (but not wild-type cells) was observed, and then incubated for 3 days. The mutants showing resistance to the low pH conditions were isolated, and the sites of transposon insertion were identified as described previously^72,73^.

### Lipid extraction and TLC analysis

Lipids were extracted from *S. cerevisiae* as described previously^74^ with minor modifications. Briefly, cells (3 *A*_600_ U (for detection of complex sphingolipids or glycerophospholipids), 5 *A*_600_ U (for detection of Cer-C), or 1.5 *A*_600_ U (for detection of ergosterol)) were suspended in 350 µl of ethanol/water/diethyl ether/pyridine/15 M ammonia (15:15:5:1:0.018, v/v), and then incubated at 65 °C for 15 min. The lipid extracts were centrifuged at 10,000*g* for 1 min and then extracted once more in the same manner. The resulting supernatants were dried. For analysis of complex sphingolipids and Cer-C, the lipid extracts were dissolved in 130 µl monomethylamine (40% methanol solution)/water (10:3, v/v), incubated for 1 h at 53 °C (mild alkaline treatment), and then dried. The lipids were suspended in 60 µl of chloroform/methanol/water (5:4:1, v/v), and then separated on Silica Gel 60 TLC plates (Merck, Whitehouse Station, NJ, USA) with chloroform/methanol/4.2 M ammonia (9:7:2, v/v) (for detection of complex sphingolipids), chloroform/methanol/acetic acid (100:6:0.6, v/v) (for detection of Cer), or hexane/diethyl ether/acetic acid (30:70:1, v/v) (for detection of ergosterol) as the solvent system. Glycerophospholipids were separated on a LK5 silica gel 150A TLC plate (Whatman, Clifton, NJ), which had been pre-washed in chloroform/methanol (1:1, v/v) and treated with 2% boric acid in ethanol. The TLC plates were developed two times with chloroform/ethanol/water/triethylamine (30:35:7:35, v/v)^75^. The TLC plates were sprayed with 10% copper sulphate in 8% orthophosphoric acid and then heated at 180 °C to visualize lipids. Identification of each complex sphingolipid, Cer-C, glycerophospholipid, and ergosterol was performed as described in previous papers^8,37,73,75^. The relative amounts of lipids were determined with ImageJ software (National Institutes of Health, Bethesda, MD, USA).

### Quantification of sphingolipids by HPLC analysis

HPLC analysis of sphingolipids was performed as described previously^2,40,76^ with some modifications. Yeast cells (2 *A*_600_ U) were collected by centrifugation and then washed with water. After the addition of 1 nmol of sphingosine (d18:1) (Biomol, Plymouth Meeting, PA, USA) as an internal standard, lipids were extracted as described above. For acid hydrolysis, the lipids were dissolved in 500 μl of methanol/water (82:18, v/v) containing 1 M HCl, and then heated at 80 °C for 18 h. After the addition of 500 μl of 3 M NH_4_OH, the hydrolyzed LCBs were extracted two times with 500 μl of chloroform. The combined chloroform extracts were washed with 300 μl of 3 M NH_4_OH three times, dried, and then dissolved in 120 μl of ethanol by heating at 67 °C for 25 min. The lipid solution was mixed with 15 μl of OPA (*o*-phthalaldehyde) reagent (1 mg of OPA, 20 μl of ethanol, 2 µl of 2-mercaptoethanol, and 1 ml of a 3%, w/v, boric acid solution adjusted to pH 10.5), followed by incubation at room temperature for 30 min. Samples were centrifuged at 10,000*g* for 5 min, and the resulting supernatants were incubated overnight at room temperature. Then, the samples were resolved by HPLC on a pre-packed C_18_ reversed-phase column (Cosmosil 5C18-AR-II; Nacalai Tesque, Kyoto, Japan) using an isocratic eluent composition of acetonitrile/water (90:10, v/v) and a flow rate of 1 ml/min. The OPA derivatives were monitored at an excitation wavelength of 340 nm and an emission wavelength of 455 nm. The areas of peaks of LCBs (phytosphingosine (PHS; t18:0) and dihydrosphingosine (DHS; d18:0)) were determined using sphingosine as an internal standard.

### Yeast protein extraction, SDS-PAGE, and Western blotting

Protein extraction, SDS-PAGE, and Western blotting were performed as described elsewhere^17^ with some modifications. For protein extraction, yeast cells grown in YPD medium were collected by centrifugation, washed with water, and then resuspended in 100 µl of 0.2 N NaOH containing 0.5% 2-mercaptoethanol. The suspension was incubated on ice for 15 min. One ml of ice-cold acetone was added to the suspension, followed by incubation for 30 min at -25 °C, and then the proteins were precipitated by centrifugation for 10 min at 10,000*g*. The pellet was resuspended in 100 µl of SDS sample buffer (156 mM Tris–HCl, pH 6.8, containing 5% SDS, 25% glycerol, 5% 2-mercaptoethanol and 0.001% bromophenol blue). The suspension was mixed well, heated for 3 min at 95 °C, and then centrifuged for 2 min at 10,000*g*. Then the supernatant was separated by SDS-PAGE according to the method of Laemmli^77^. For Western blotting, anti-Pma1 (40B7; Santa Cruz Biotechnology, Santa Cruz, CA, USA), anti-HA (HA-7; Sigma-Aldrich), anti-FLAG (M2; Sigma-Aldrich), and anti-Pgk1 (22C5D8; Thermo Fisher Scientific, Waltham, MA, USA) were used as primary antibodies. Horseradish peroxidase-conjugated anti-mouse IgG (Thermo Fisher Scientific) was used as the secondary antibody. The relative intensity of each protein band was determined with ImageJ software.

### SDS-PAGE on phosphate-affinity gels

Phos-tag SDS-PAGE was performed as described elsewhere^39^.

### Evaluation of intracellular acidification

Cells harboring pKL06 were cultured overnight in SC-Leu medium (pH 6.0), diluted (1 *A*_600_ units/ml) in fresh YPD medium, and then incubated for 3 h at 30 °C. The cells were resuspended in fresh YPD medium with or without 30 or 150 mM acetic acid, which was buffered to pH 4.0, 5.5, or 3.7, to 0.7 *A*_600_ units/ml and then incubated for 1 h at 30 °C. The cells were viewed under a fluorescence microscope. The fluorescence intensities of super*-*ecliptic pHluorin and mRuby2 excited at 475 nm and 555 nm, respectively, were quantified with ImageJ software.

### Rhodamine-6G staining

Cells were cultured overnight in YPD medium, diluted (1 *A*_600_ units/ml) in fresh YPD medium, and then incubated for 3 h at 30 °C. The cells were resuspended in fresh YPD medium buffered to pH 5.5 or 3.7 to 0.7 *A*_600_ units/ml, and then incubated for 1.5 h at 30 °C. Then, 10 µM rhodamine 6G was added to the culture medium, followed by incubation for 30 min at 30 °C. The cells were collected by centrifugation, washed two times with water, and then viewed under a fluorescence microscope. The fluorescence intensity was quantified with ImageJ software.

### Filipin staining

Cells were grown to the exponential phase, and then filipin was added to the medium at a final concentration of 15 µg/ml. Cells were observed immediately under a fluorescence microscope. The fluorescence intensity was quantified with ImageJ software.

### Statistical analysis

Statistical analysis was done using Student’s t test, and the *P* values obtained are indicated.

## Supplementary information

Supplementary file1 (PDF 9687 kb)

## Abbreviations

AmB: Amphotericin B
Cer: Ceramide
DHS: Dihydrosphingosine
Dox: Doxycycline
IPC: Inositol phosphorylceramide
LAM: Lipid transfer protein anchored at a membrane contact site
LCB: Long chain base
MIPC: Mannosylinositol phosphorylceramide
M(IP)_2_C: Mannosyldiinositol phosphorylceramide
PHS: Phytosphingosine
SPT: Serine palmitoyltransferase
