# Supplementary material for: Proper regulation of inositolphosphorylceramide levels is required for acquirement of low pH resistance in budding yeast
Source: Sci Rep. 2020 Jul 1;10:10792. doi: 10.1038/s41598-020-67734-8 (PMC7329899; doi:10.1038/s41598-020-67734-8)
Supplement: Supplementary file 1 — Supplementary file1 (PDF 9687 kb) [file 41598_2020_67734_MOESM1_ESM.pdf]

SUPPLEMENTARY INFORMATION

Proper regulation of inositolphosphorylceramide levels is required for acquirement of low pH resistance in the budding yeast

Mikiko Otsu[1], Moeko Toume[1], Yutaro Yamaguchi[1], and Motohiro Tani[1]§

[1]Department of Chemistry, Faculty of Sciences, Kyushu University, 744, Motooka, Nishi-ku, Fukuoka 819-0395, Japan

§ Corresponding author: Department of Chemistry, Faculty of Sciences, Kyushu University, 744, Motooka, Nishi-ku, Fukuoka 819-0395, Japan.
Tel: +81-92-802-4181; Fax: +81-92-802-4181; E-mail: tani@chem.kyushu-univ.jp

Table S1. Genes identified on transposon mutagenesis and their known functions.

Gene	No.[a]	Function
SIP3	1	Protein involved in retrograde transport of sterols from plasma membranes to the ER
LAM1	1	Protein involved in retrograde transport of sterols from plasma membranes to the ER
PMR1	1	Ca^{2+}/Mn^{2+} P-type ATPase
XRN1	2	5'-3'-exonuclease
LCB4	1	LCB kinase
SUR2	1	Sphingolipid C4-hydroxylase

a. Number of transposon-inserted mutants isolated this study.

Table S2. Strains used in this study.

Strain	Genotype	Source
BY4741	*MATa his3Δ1 leu2Δ0 met15Δ0 ura3Δ0*	1
STY39	BY4741, *sur1Δ::natMX4 csh1Δ::hphNT1*	2
MTY1467	BY4741, *sur1Δ::kanMX4*	This study
MTY1466	BY4741, *csh1Δ::kanMX4*	This study
MTY1468	BY4741, *csg2Δ::kanMX4*	This study
MTY1469	BY4741, *ipt1Δ::kanMX4*	3
MTY215	BY4741, *sur2Δ::kanMX4*	3
MTY1253	BY4741, *scs7Δ::hphNT1*	3
MTY642	BY4741, *elo3Δ::natMX4*	This study
MTY157	BY4741, *elo2Δ::URA3*	This study
MTY2395	BY4741, *ADHp-PMA1::kanMX4*	This study
MTY2396	BY4741, *sur1Δ::natMX4 csh1Δ::hphNT1 ADHp-PMA1::kanMX4*	This study
MTY2407	BY4741, *pdr5Δ::kanMX4*	This study
MTY2408	BY4741, *sur1Δ::natMX4 csh1Δ::hphNT1 pdr5Δ::kanMX4*	This study
MTY2292	BY4741, *sur1Δ::natMX4 csh1Δ::hphNT1 sip3Δ::kanMX4*	This study
MTY2295	BY4741, *sur1Δ::natMX4 csh1Δ::hphNT1 lam1Δ::kanMX4*	This study
MTY2293	BY4741, *sur1Δ::natMX4 csh1Δ::hphNT1 pmr1Δ::kanMX4*	This study
MTY2294	BY4741, *sur1Δ::natMX4 csh1Δ::hphNT1 xrn1Δ::kanMX4*	This study
MTY2296	BY4741, *sur1Δ::natMX4 csh1Δ::hphNT1 lcb4Δ::kanMX4*	This study
MTY2308	BY4741, *sur1Δ::natMX4 csh1Δ::hphNT1 sur2Δ::kanMX4*	This study
MTY2313	BY4741, *sur1Δ::natMX4 csh1Δ::hphNT1 scs7Δ::kanMX4*	This study
MTY2297	BY4741, *sip3Δ::kanMX4*	This study
MTY2300	BY4741, *lam1Δ::kanMX4*	This study
MTY2298	BY4741, *pmr1Δ::kanMX4*	This study
MTY2299	BY4741, *xrn1Δ::kanMX4*	This study
MTY2301	BY4741, *lcb4Δ::kanMX4*	This study

MTY59	BY4741, *tetO7-LIP1::kanMX4*	4
MTY2315	BY4741, *tetO7-LIP1::kanMX4 sur1Δ::natMX4 csh1Δ::hphNT1*	This study
MTY1402	BY4741, *LCB1-6xHA::hphNT1*	5
MTY1316	BY4741, *AUR1-6xHA::hphNT1*	3
MTY1492	BY4741, *KEI1-6xHA::hphNT1*	This study
MT57	BY4741, *ORM2-6xHA::hphNT1*	5
MT55	BY4741, *ORM1-6xHA::hphNT1*	4
MTY1389	BY4741, *3xFLAG-LAG1::hphNT1*	This study
MTY1390	BY4741, *3xFLAG-LAC1::hphNT1*	This study
MT2	BY4741, *orm1Δ::kanMX4*	5
MT4	BY4741, *orm2Δ::kanMX4*	5
MT27	BY4741, *orm1Δ::kanMX4 orm2Δ::natMX4*	5
YY17	BY4741, *TEFp-AUR1::natNT2*	This study
MTY2342	BY4741, *TEFp-AUR1-6xHA::natNT2, hphNT1*	This study
MTY2364	BY4741, *TEFp-AUR1::natNT2 orm1Δ::LEU2 orm2Δ::URA3*	This study
MTY2331	BY4741, *sur1Δ::natMX4 csh1Δ::hphNT1 ysp2Δ::kanMX4*	This study
MTY2332	BY4741, *sur1Δ::natMX4 csh1Δ::hphNT1 lam4Δ::kanMX4*	This study
MTY2333	BY4741, *sur1Δ::natMX4 csh1Δ::hphNT1 lam5Δ::kanMX4*	This study
MTY2334	BY4741, *sur1Δ::natMX4 csh1Δ::hphNT1 lam6Δ::kanMX4*	This study
MTY2337	BY4741, *sur1Δ::natMX4 csh1Δ::hphNT1 sip3Δ::kanMX4 lam1Δ::URA3 ysp2Δ::LEU2*	This study
STY81	BY4741, *erg2Δ::kanMX4*	2
STY83	BY4741, *erg2Δ::kanMX4 sur1Δ::natMX4 csh1Δ::hphNT1*	2
STY82	BY4741, *erg3Δ::kanMX4*	2
STY84	BY4741, *erg3Δ::kanMX4 sur1Δ::natMX4 csh1Δ::hphNT1*	2
STY77	BY4741, *erg4Δ::kanMX4*	2
STY79	BY4741, *erg4Δ::kanMX4 sur1Δ::natMX4 csh1Δ::hphNT1*	2
STY78	BY4741, *erg5Δ::kanMX4*	2
MTY2297	BY4741, *sip3Δ::kanMX4*	This study
MTY2300	BY4741, *lam1Δ::kanMX4*	This study
MTY2338	BY4741, *ysp2Δ::kanMX4*	This study
MTY2339	BY4741, *lam4Δ::kanMX4*	This study
MTY2340	BY4741, *lam5Δ::kanMX4*	This study
MTY2341	BY4741, *lam6Δ::kanMX4*	This study
MTY2424	BY4741, *ypk1-ts::HIS3 ypk2Δ::kanMX4*	6

Table S3. DNA primers used in this study.

Primer name	Sequence (5’-3’)
LAG1-3xFLAG-HindIII-F	TAAAAGCTTACATCAGCTACGGACAAATCTA
LAG1-3xFLAG-BamHI-R	AAGGATCCTTATTCACACTTTTCCTTAGATTCTTCA
LAC1-3xFLAG-HindIII-F	TAAAAGCTTTCGACAATAAAGCCAAGCCCTT
LAC1-3xFLAG-BamHI-R	AAGGATCCTCAAATATCCTTTTTCGTTGGAGTA
3xFLAG-LAG1-F1	TGAGAGTGAACTCCAAGATACAGAGAAACTGAAGAAATAACGACAACATGGACTACAAAGACCATGACGG
LAG1Hyg-R	GTTCCACTTTTTATTATTCACACTTTTCCTTAGATTCTTCA
3xFLAG-LAC1-F1	ACCTCCGGTAAACATTTAGATAGACACAGTATCAATAAACAAGAGCTATGGACTACAAAGACCATGACGG
LAC1Hyg-R	GTTCCACTTTTTATCAAATATCCTTTTTCGTTGGAGTA
LAG1-S2	TACAGGGGGGAAATCATATGATGATACGTATTCTCCTTAAGATACGTTTAATCGATGAATTCGAGCTCG
LAG1Hyg-F	AAAAGTGTGAATAATAAAAAGTGGAACGATCATTCA
LAC1-S2	AAGAATTAATGTGTAATGGTTATACTACTTAAAAACACCGTTTTCCTTCAATCGATGAATTCGAGCTCG
LAC1Hyg-F	AAAAAGGATATTTGATAAAAAGTGGAACGATCATTCA
SUR1-6HA-SacI-S	AAAGAGCTCCACGCTTGCTGCGTCTCTT
SUR1-6HA-KpnI-A	TTGGGTACCGGGGACGAGGCAAGCTAAAC

Underlining indicates restriction enzyme sites.

REFERENCES

1 Brachmann, C. B. *et al.* Designer deletion strains derived from Saccharomyces cerevisiae S288C: a useful set of strains and plasmids for PCR-mediated gene disruption and other applications. *Yeast* **14**, 115-132, doi:10.1002/(SICI)1097-0061(19980130)14:2<115::AID-YEA204>3.0.CO;2-2 (1998).

2 Tanaka, S. & Tani, M. Mannosylinositol phosphorylceramides and ergosterol coodinately maintain cell wall integrity in the yeast Saccharomyces cerevisiae. *FEBS J* **285**, 2405-2427, doi:10.1111/febs.14509 (2018).

3 Tani, M. & Toume, M. Alteration of complex sphingolipid composition and its physiological significance in yeast Saccharomyces cerevisiae lacking vacuolar ATPase. *Microbiology* **161**, 2369-2383, doi:10.1099/mic.0.000187 (2015).

4 Toume, M. & Tani, M. Yeast lacking the amphiphysin-family protein Rvs167 are sensitive to disruptions in sphingolipid levels. *FEBS J* **283**, 2911-2928, doi:10.1111/febs.13783 (2016).

5 Toume, M. & Tani, M. Change in activity of serine palmitoyltransferase affects sensitivity to syringomycin E in yeast Saccharomyces cerevisiae. *FEMS Microbiol Lett* **358**, 64-71, doi:10.1111/1574-6968.12535 (2014).

6 Arita, N., Sakamoto, R. & Tani, M. Mitochondrial reactive oxygen species-mediated cytotoxicity of intracellularly accumulated dihydrosphingosine in the yeast Saccharomyces cerevisiae. *FEBS J*, doi:10.1111/febs.15211 (2020).

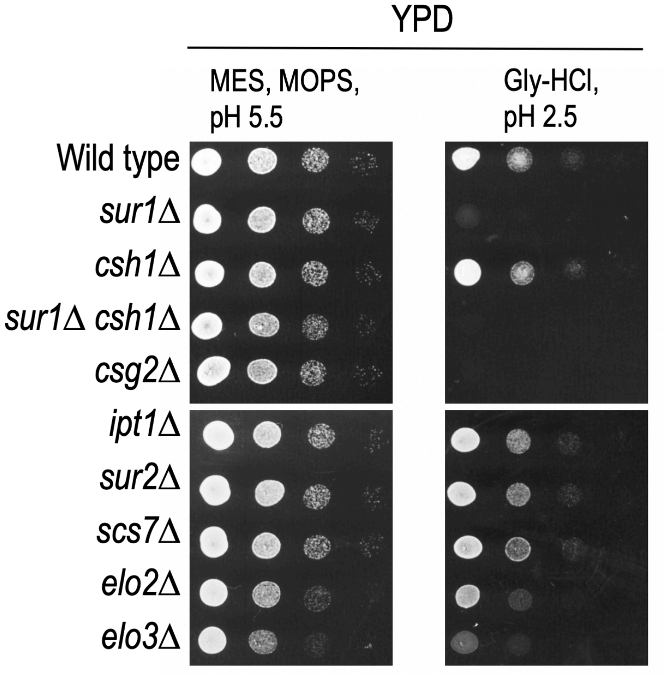

Figure S1. YPD plates showing growth of sphingolipid-metabolizing enzyme-deleted yeast cells after 1 day.

Cells were cultured overnight in YPD medium at 30°C, and then spotted onto agar plates containing YPD medium buffered with 50 mM MES and 50 mM MOPS (for pH 5.5), or 100 mM glycine-HCl (for pH 2.5) in 10-fold serial dilutions starting with a density of 0.7 A_{600} units/ml. All plates were incubated at 30°C and photographed after 1 day.

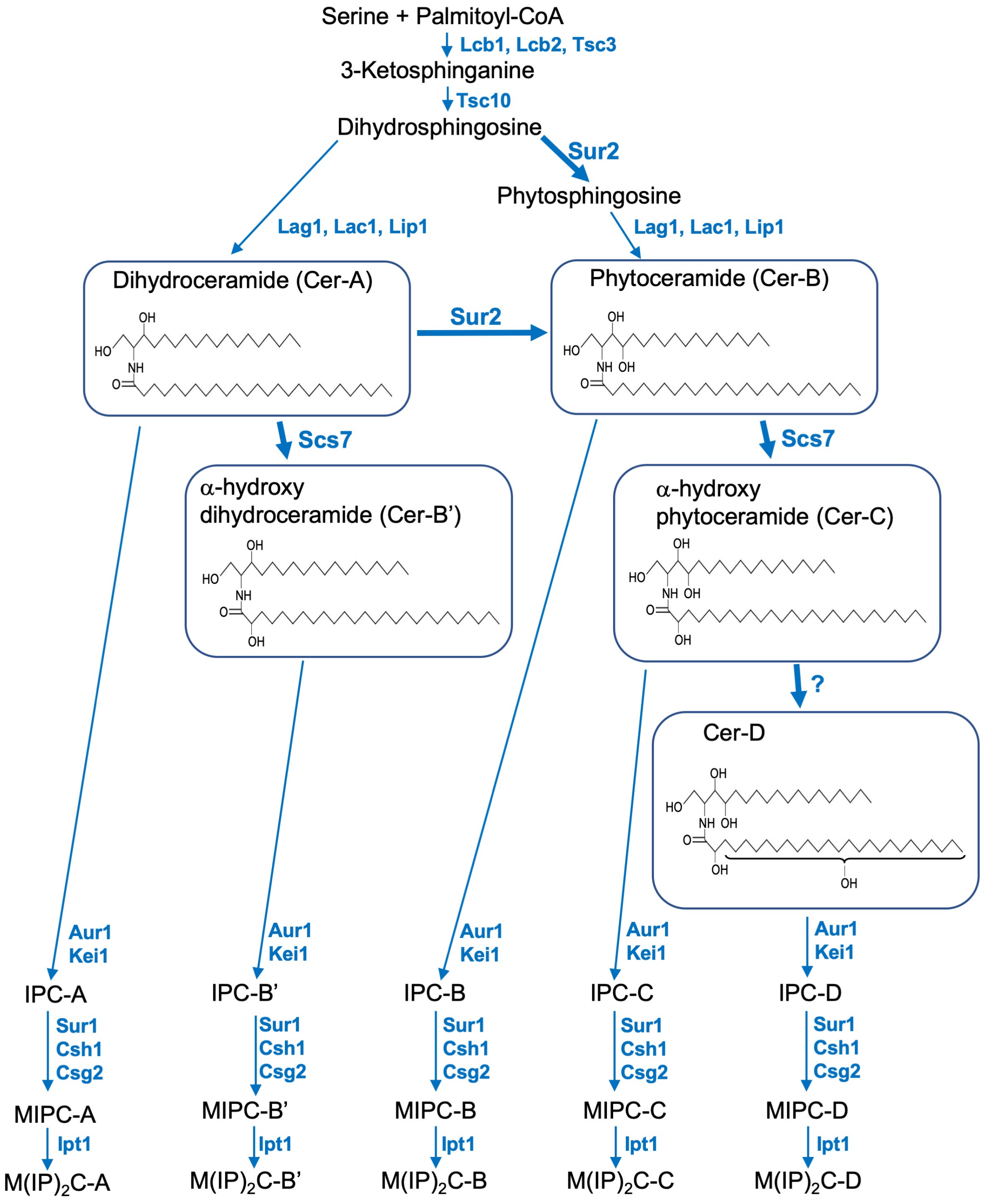

Figure S2. Overview of hydroxylation pathway of *S. cerevisiae* sphingolipids.

In yeast, Cers and complex sphingolipids can be divided in five types (A, B, B', C and D) according to the hydroxylation state. The hydroxylation of sphingolipids is catalyzed by Sur2, Scs7, and an unidentified hydroxylase(s). Each hydroxylation step is indicated as thick arrows.

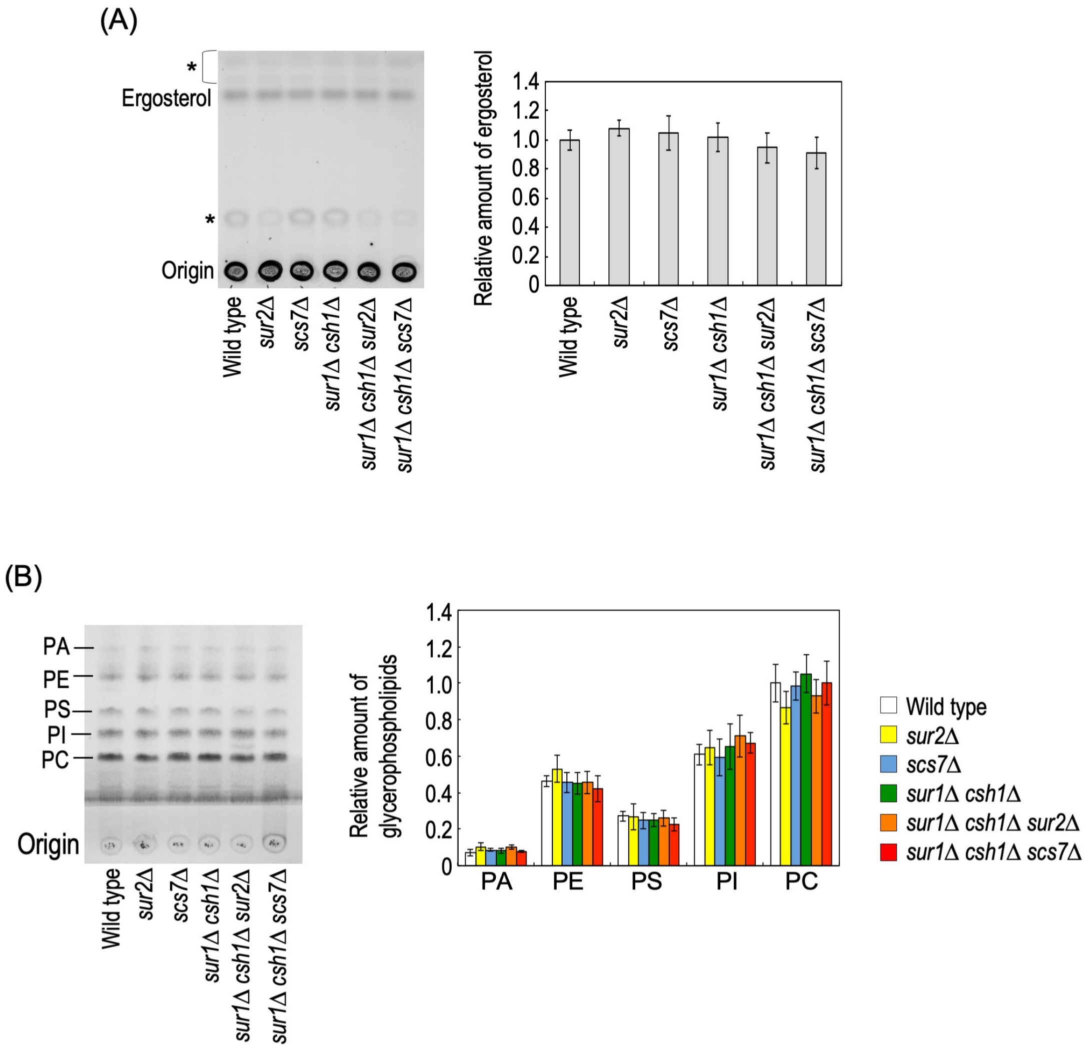

Figure S3. Ergosterol and glycerophospholipid levels in *SUR1*-, *CSH1*-, *SUR2*-, or *SCS7*-deleted cells.

Wild-type cells were cultured overnight in YPD medium, diluted (0.3 A_{600} units/ml) in fresh YPD medium, and then incubated for 5 h at 30°C. Lipids were extracted and then separated by TLC. The lipids were visualized with a copper sulfate and orthophosphoric acid reagent. The asterisks indicate unidentified bands. The details are given under METHODS. Relative amounts of ergosterol and glycerophospholipids (phosphatidylcholine (PC), phosphatidylinositol (PI), phosphatidylserine (PS), phosphatidylethanolamine (PE), and phosphatidic acid (PA)) were determined with ImageJ software. The amount of ergosterol (A) or PC (B) in wild-type cells was taken as 1. Data represent means ± SD for one experiment (triplicate) representative of three independent experiments.

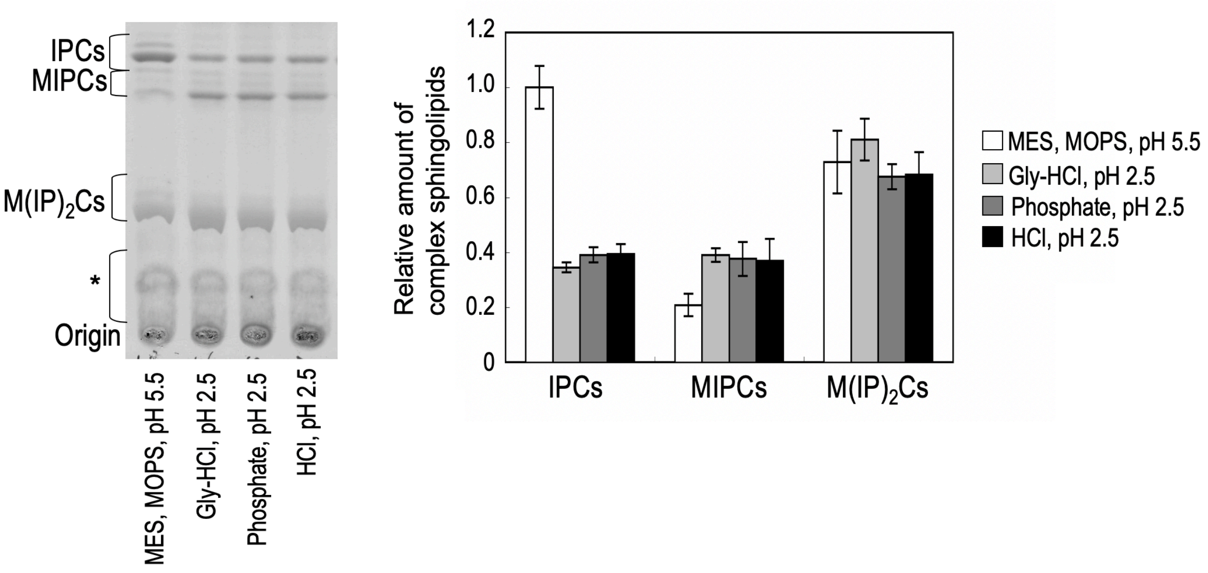

Figure S4. Complex sphingolipid analysis of cells incubated at pH 2.5.

Wild-type cells were cultured overnight in YPD medium, diluted (0.7 A_{600} units/ml) in fresh YPD medium, and then incubated for 3.5 h at 30°C. The cells were resuspended in fresh medium buffered with 50 mM MES and 50 mM MOPS (for pH 5.5), 100 mM glycine-HCl (for pH 2.5), or 100 mM phospholic acid-sodium dihydrogen phosphate (for pH 2.5) to 0.5 A_{600} units/ml, and then incubated for 3 h at 30°C. YPD medium (pH 2.5) was also prepared by the addition of HCl. Lipids were extracted, treated with monomethylamine, and then separated by TLC. The lipids were visualized with a copper sulfate and orthophosphoric acid reagent. The relative amounts of complex sphingolipids were determined with ImageJ software. The amount of IPCs in wild-type cells at pH 5.5 was taken as 1. The asterisk indicates unidentified bands. Data represent means ± SD for one experiment (triplicate) representative of three independent experiments.

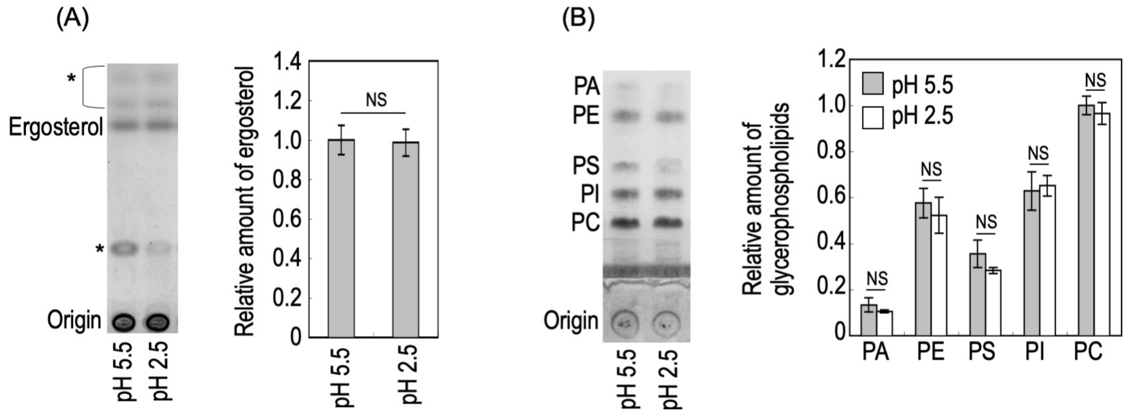

Figure S5. TLC analysis of ergosterol and glycerophospholipids.

Wild-type cells were cultured overnight in YPD medium, diluted (0.7 A_{600} units/ml) in fresh YPD medium, and then incubated for 3.5 h at 30°C. The cells were resuspended in fresh YPD medium buffered to pH 5.5 or 2.5 to 0.5 A_{600} units/ml, and then incubated for 3 h at 30°C. Lipids were extracted and then separated by TLC. The lipids were visualized with a copper sulfate and orthophosphoric acid reagent. The asterisks indicate unidentified bands. The details are given under METHODS. The relative amounts of ergosterol and glycerophospholipids (PC, PI, PS, PE and PA) were determined with ImageJ software. The amount of ergosterol (A) or PC (B) in wild-type cells at pH 5.5 was taken as 1. Data represent means ± SD for one experiment (triplicate) representative of three independent experiments. Statistical analysis was done using Student's t test. NS, no significant difference.

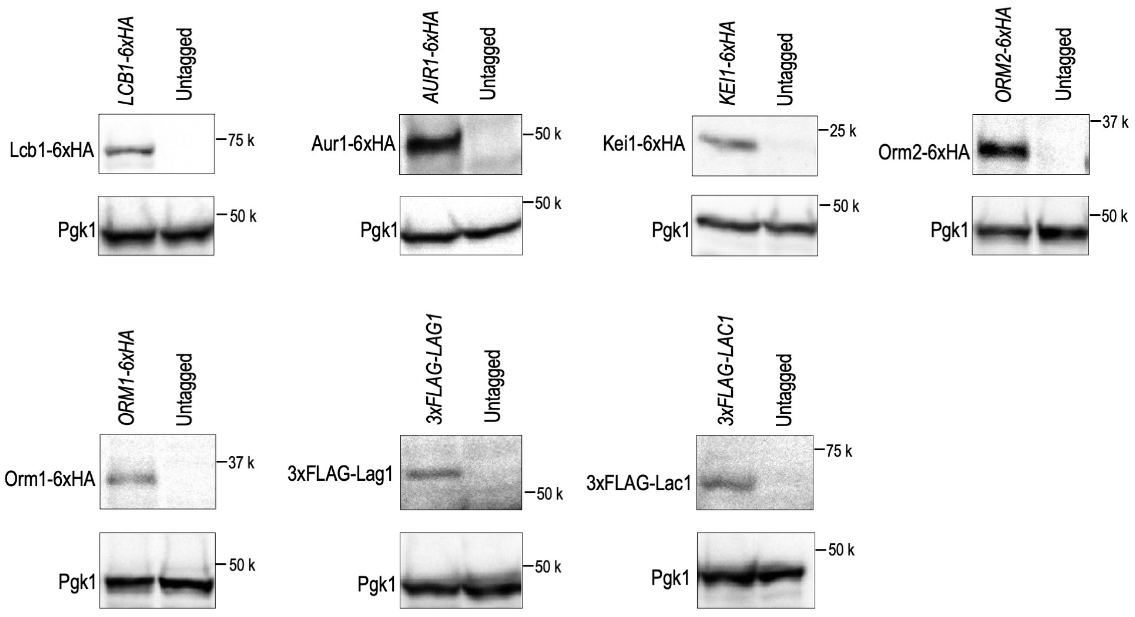

Figure S6. Confirmation of expression of proteins tagged with 6xHA or 3xFLAG.

Cells expressing proteins tagged with or without 6xHA or 3xFLAG were cultured to the exponential growth, and then cell extracts were immunoblotted using anti-HA, anti-FLAG or anti-Pgk1. The details are given under METHODS. Full Western blots are shown in Fig. S11, *panel a.*

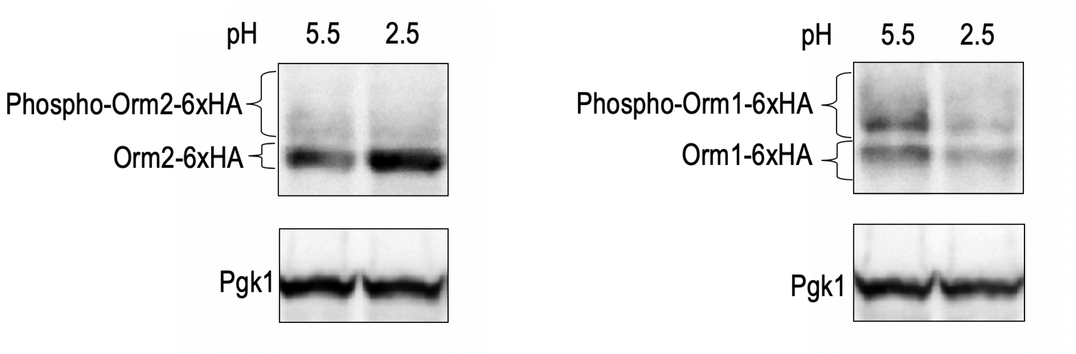

Figure S7. Analysis of phosphorylation of Orm2-6xHA and Orm1-6xHA.

Cells expressing Orm2-6xHA or Orm1-6xHA were cultured overnight in YPD medium, diluted (0.7 A_{600} units/ml) in fresh YPD medium, and then incubated for 3.5 h at 30°C. The cells were resuspended in fresh YPD medium buffered to pH 5.5 or 2.5 to 0.5 A_{600} units/ml, and then incubated for 3 h at 30°C. Yeast cell extracts were separated by phos-tag SDS-PAGE, and then immunoblotted using anti-HA or anti-Pgk1 antibodies. The details are given under METHODS. Full Western blots are shown in Fig. S11, *panel b*.

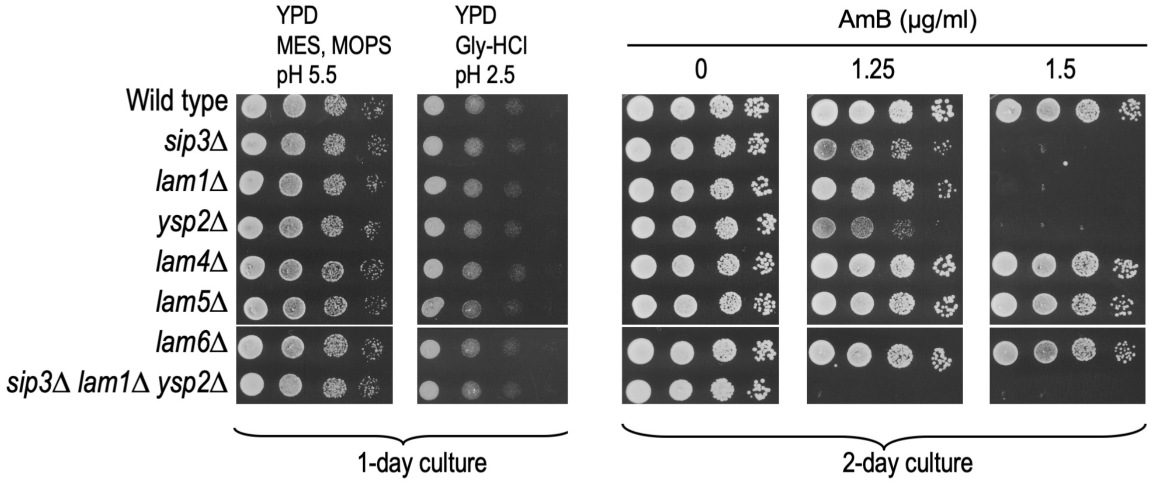

Figure S8. AmB and pH sensitivities of cells lacking LAM family proteins.

Cells were cultured overnight in YPD medium at 30°C, and then spotted onto agar plates containing YPD medium buffered to 5.5 or 2.5, or YPD medium (pH 6.0) containing the indicated amounts of AmB in 10-fold serial dilutions starting with a density of 0.7 A_{600} units/ml. All plates were incubated at 30°C and photographed after 1 day or 2 days.

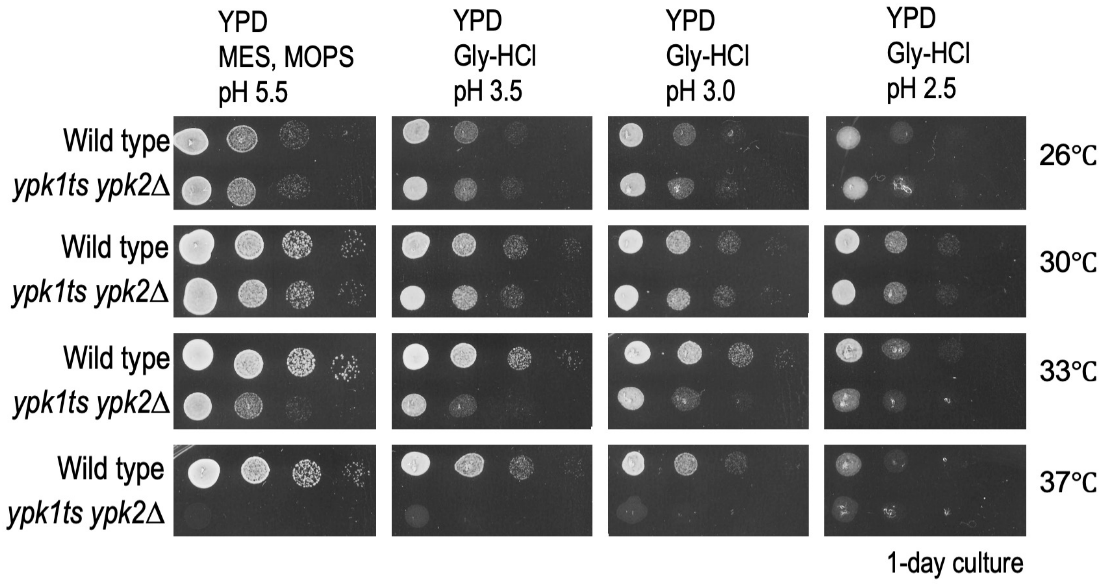

Figure S9. Low pH sensitivity of *ypk1ts ypk2*Δ cells.

Wild-type and *ypk1ts ypk2*Δ cells were cultured overnight in YPD medium at 26°C, and then spotted onto agar plates containing YPD medium buffered to 5.5, 3.5, 3.0, or 2.5 in 10-fold serial dilutions starting with a density of 0.7 A_{600} units/ml. All plates were incubated at 26, 30, 33, or 37°C and photographed after 1 day.

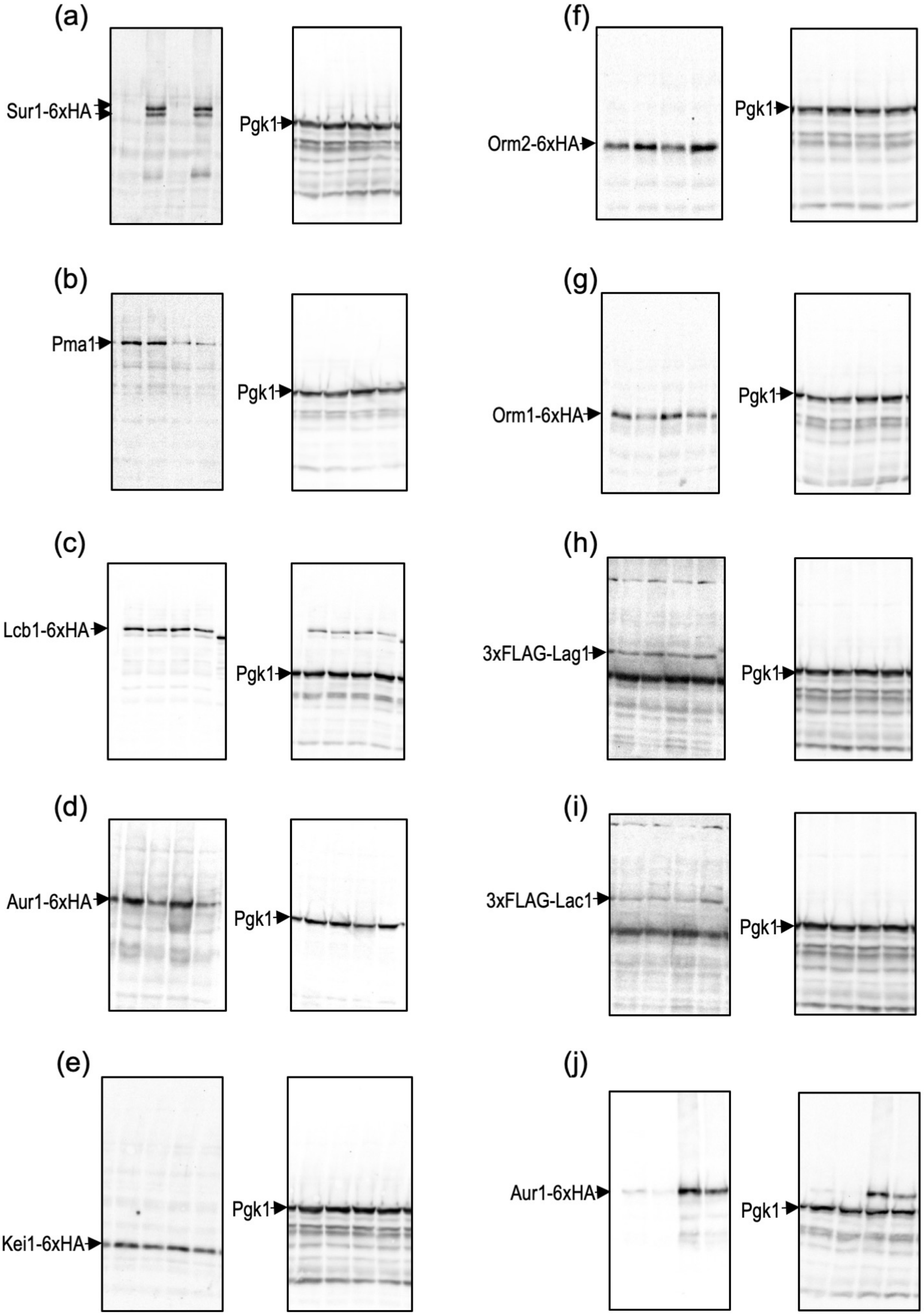

Figure S10. Full Western blots from Fig. 1F (a), Fig. 2B (b), Fig. 6F (c-i), and Fig. 7A (j).

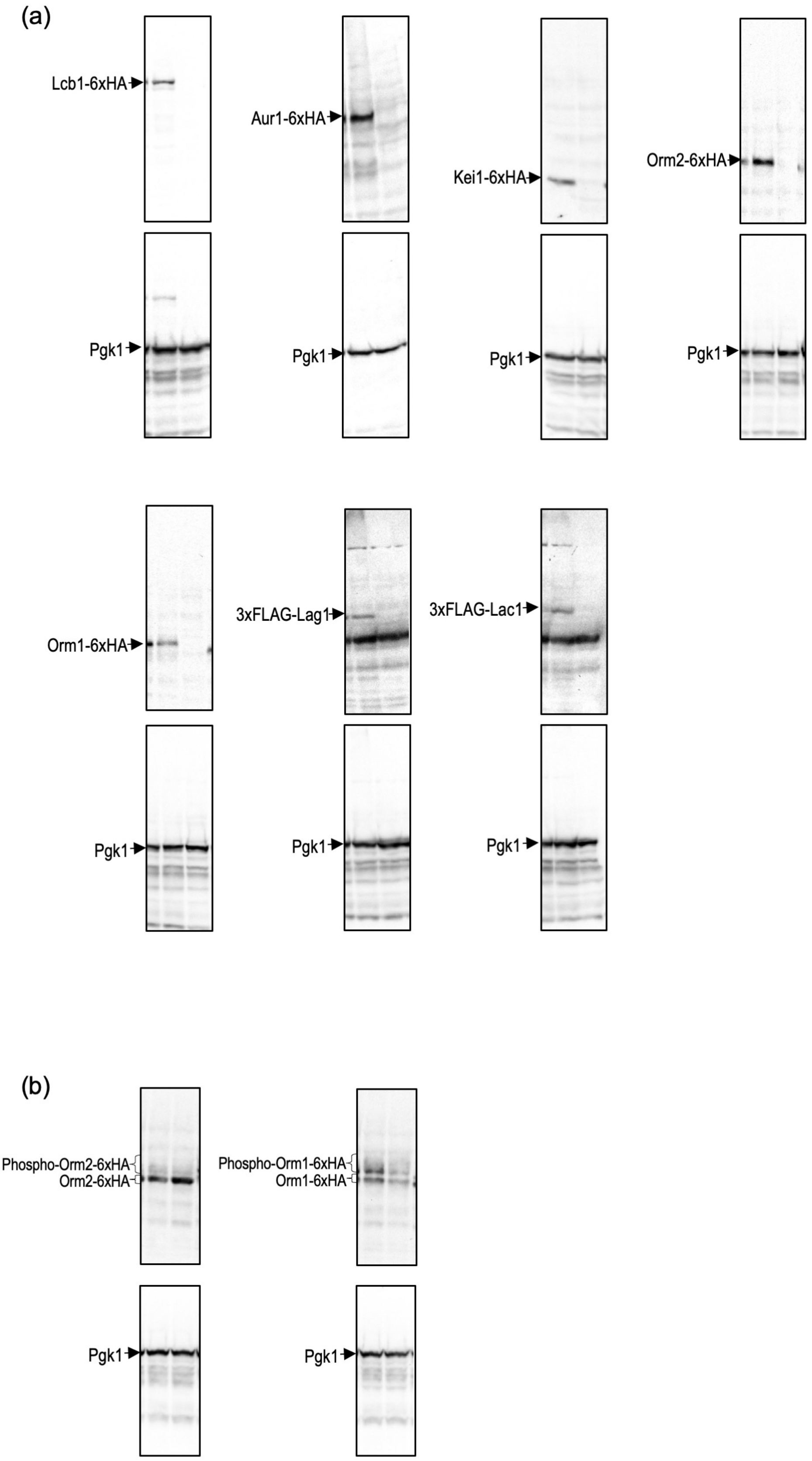

Figure S11. Full Western blots from Fig. S6 (a) and Fig. S7 (b).
